# Cysteine oxidation of a redox hub within complex I can facilitate electron transport chain supercomplex formation

**DOI:** 10.1016/j.jbc.2025.110555

**Published:** 2025-08-05

**Authors:** Runtai Chen, Seyed Amirhossein Tabatabaei Dakhili, Rokas Gerulskis, Yuan-Yuan Zhao, Steven Lockhart, Lusine Tonoyan, Arno G. Siraki, Guocheng Huang, Adam Kinnaird, Darren H. Freed, Shelley D. Minteer, Evangelos D. Michelakis, John R. Ussher, Gopinath Sutendra

**Affiliations:** 1 Department of Medicine, University of Alberta, Edmonton, Alberta, Canada; 2 Cardiovascular Research Institute, University of Alberta, Edmonton, Alberta, Canada; 3 Cancer Research Institute of Northern Alberta, University of Alberta, Edmonton, Alberta, Canada; 4 Faculty of Pharmacy and Pharmaceutical Sciences, University of Alberta, Edmonton, Alberta, Canada; 5 Department of Chemistry, University of Utah, Salt Lake City, Utah, USA; 6 Applied Pharmaceutical Innovation, University of Alberta, Edmonton, Alberta, Canada; 7 Division of Urology, Department of Surgery, University of Alberta, Edmonton, Canada; 8 Division of Cardiac Surgery, Department of Surgery, University of Alberta, Edmonton, Canada; 9 Department of Chemistry, Missouri University of Science and Technology, Rolla, Missouri, USA

**Keywords:** electron transport chain, mitochondria, reactive oxygen species, cysteine oxidation, supercomplex, cancer resistance, respirasome, redox

## Abstract

The mitochondrial electron transport chain (ETC) is a four complex unit that could be considered the most essential infrastructure within the mitochondria, as it primarily functions to generate the mitochondrial membrane potential (ΔΨm), which can then be utilized for ATP synthesis or heat production. Another important aspect of ETC function is the generation of mitochondrial reactive oxygen species (mtROS), which are essential physiologic signaling mediators that can be toxic to the cell if their levels become too high. Currently, it remains unresolved how a highly utilized and functioning ETC can sense excessive mtROS generation and adapt, to enhance ΔΨm. Here, we identified a redox hub consisting of cysteine (Cys) residues 64, 75, 78, and 92 within Ndufs1 of complex I of the ETC. Oxidation of these Cys residues promotes the incorporation of complex I into the respirasome supercomplex. Mechanistically, oxidation of the redox hub increased the distance between Fe-S clusters N5 and N6a in complex I, compromising complex I activity. This impairment was rescued by integration with complex III_2_ and IV into the respirasome supercomplex. Compared to parental cells or Ndufs1-KO cells, C92D (an oxidation mimetic) Ndufs1-knockin A549 cells had higher levels of ETC supercomplexes, ΔΨm and oxygen consumption rates, while isolated mitochondrial membranes generated more electrical current when integrated onto a biobattery platform. Disruption of ETC supercomplexes with MitoTam increased the therapeutic efficacy of mtROS inducing chemotherapeutics in both C92D Ndufs1-knockin or metastatic lung cancer cells. These findings provide new insights into how the ETC can initiate supercomplex transformation.

The mitochondrial electron transport chain (ETC) could be viewed as a biochemical battery, where complexes I or II receive electrons from NADH or succinate, respectively, before they are transported through various iron-sulfur (Fe-S) clusters to aid in the generation of an electrochemical gradient and are lastly transferred to oxygen, the terminal electron acceptor, which is reduced to water (1, 2). The resultant mitochondrial membrane potential (ΔΨm, which equates to energy capacity) can now be used by ATP synthase to generate ATP (from ADP) or by uncoupling proteins to generate heat or even for the transport of proteins into the mitochondria (1, 2, 3).

A separate, yet essential function for a highly utilized ETC is the generation of superoxide, due to electron leak from complex I and/or III, which can then be converted to the more stable hydrogen peroxide (H_2_O_2_) by manganese superoxide dismutase (2, 4, 5). H_2_O_2_ is an important biological signaling molecule that can diffuse from the mitochondria and travel to distant parts of the cell (including cell membranes or the nucleus), to facilitate cysteine oxidative posttranslational modifications (*i*.*e*., disulfide bonds, sulfenylation, sulfinylation, or sulfonation) (6, 7, 8). Alternatively, if mitochondrial superoxide (and subsequently H_2_O_2_ levels) become excessive, it can have detrimental effects on the cell, which can include DNA damage and subsequent DNA mutations, resulting in cell death or initiation of pathological conditions like cancer (9). Additionally, excessive ETC electron leak (during forward electron transfer (5, 10)) can result in a less efficient electrochemical gradient, and subsequently a suboptimal ΔΨm (3, 11). Although under normal conditions the amount of electron leak to superoxide (within the ETC) is minimal (1–2% of overall oxygen consumption) (12), in a highly utilized ETC (*i*.*e*., cells that have a high demand for ATP) and over a given timeframe this could result in large amounts of lost or wasted energy (in the form of prospective ΔΨm). What remains unresolved is how the ETC can sense excessive mitochondrial reactive oxygen species (mtROS) and adapt, to efficiently transfer electrons and subsequently optimize ΔΨm. This type of biological “battery” would be desirable to cells that primarily utilize the ETC for oxidative phosphorylation and require large amounts of ATP for cell function, including cardiomyocytes (for contractile force) (13) or metastatic cancer cells (for cell motility/migration) (14, 15).

It has previously been reported that the mitochondrial ETC complexes I, III, and IV (CI, CIII, and CIV) can reassemble into supercomplexes (CI_n_ + CIII_n_, CIII_n_ + CIV_n_), including the respirasome (CI_n_ + III_n_ + IV_n_) (16, 17, 18). This structural reorganization has been suggested to increase electron transfer efficiency (although somewhat controversial), minimizing the production of mtROS (19, 20, 21). It appears that CI, the first and largest complex within the ETC, is essential for the respirasome assembly (22, 23, 24). Furthermore, the NADH dehydrogenase Fe-S protein 1 (Ndufs1), the largest subunit of CI, contains three Fe-S clusters in its N-module, where it binds and oxidizes NADH (25). Deficiency or mutations in Ndufs1 has been reported to increase mtROS production and impair ΔΨm (21, 26, 27, 28), potentially due to supercomplex disassembly (21). Here, we hypothesize that a conserved redox hub exists within CI, and specifically within Ndufs1, where receptive Cys residues could be oxidized by mitochondrial H_2_O_2_. Oxidation of this redox hub in complex I may initiate a structural shift toward its integration into the efficient electron-transferring respirasome supercomplex.

## Results

### Oxidation of a redox hub within Ndufs1 of complex I can facilitate its incorporation into the respirasome supercomplex

**Figure 1:**
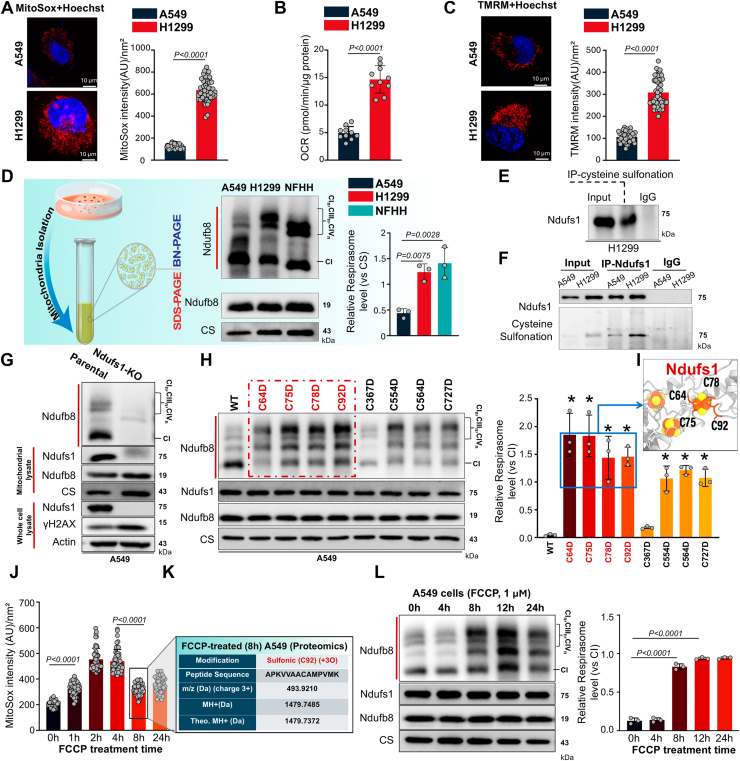
**Identification of a redox hub within Ndufs1 that can directly facilitate the transformation toward the ETC respirasome**. *A*, mitochondrial ROS were analyzed in human A549 cells and H1299 cells labeled with MitoSox. Representative confocal images show significantly higher ROS levels in H1299 cells, than A549 cells. Student’s *t* test on n = 50 cells/group. AU, arbitrary unit. MitoSox (*red*) and Hoechst (*blue*). *B*, analysis of oxygen consumption rate (OCR) in A549 cells and H1299 cells were assessed by Seahorse assay, showing higher basal OCR level in H1299 cells, than A549 cells. Student’s *t* test on n = 10 cells/group. *C*, mitochondrial membrane potential (ΔΨm) assessment in A549 cells and H1299 cells using tetramethylrhodamine methyl ester (TMRM). Confocal images illustrate increased ΔΨm in H1299 cells, compared to A549 cells. Student’s *t* test on n = 50 cells/group. AU, arbitrary unit. TMRM (*red*) and Hoechst (*blue*). *D*, mitochondrial proteins were isolated from A549 cells, H1299 cells and nonfailure human heart (NFHH) tissues and analyzed *via* BN-PAGE and SDS-PAGE, followed by immunoblotting with Ndufb8 or citrate synthase (CS) antibodies, respectively, demonstrated enhanced respirasome supercomplex levels in H1299 cells and nonfailure human hearts, compared to A549 cells. One-way ANOVA with Dunnett’s multiple comparisons test on n = 3 biologically independent replicates. *E*, co-IP and immunoblot confirmation of Ndufs1 cysteine sulfonation from H1299 cells. *F*, comparative co-IP and immunoblot analysis showing Ndufs1 sulfonation in nonfailure human heart tissues and human A549 cells, n = 3 biologically independent replicates. *G*, mitochondrial proteins from A549 cells (parental and Ndufs1-KO) were separated by BN-PAGE and SDS-PAGE. Immunoblot analysis was performed with antibodies: Ndufb8 for complex I and respirasome supercomplex containing complex I with native samples; Ndufb8, Ndufs1, and CS with denatured mitochondrial lysate samples; Ndufs1, γ-H2AX, and actin with denatured whole-cell lysate samples, Student’s *t* test on n = 3 biologically independent replicates. *H*, mitochondrial proteins from A549 cells transfected with WT Ndufs1 or different versions of mutant Ndufs1 (cysteine 64, 75, 78, 92, 367, 554, 564, or 727 mutated to aspartic acid) plasmid were separated by BN-PAGE and SDS-PAGE. One-way ANOVA with Dunnett’s multiple comparisons test on n = 3 biologically independent replicates, ∗*p* < 0.05 *versus* WT. *I*, cysteine 64, 75, 78, and 92 of Ndufs1 protein are located around an Fe-S cluster inside Ndufs1. *J*, analysis of mitochondrial ROS in human A549 cells treated with 1 μM FCCP (0–24 h), using MitoSox. *K*, C92 sulfonation in Ndulfs1 was identified from FCPP-treated 8 h A549 cells utilizing mass spectrometry proteomics. The table shows the information of the precursor ion at *m/z* 493.9210 (triple charged) with the high mass accuracy for protonated molecular ion MH^+^. *L*, BN-PAGE and SDS-PAGE separation of mitochondrial proteins from A549 cells treated with 1 μM FCCP (0–24 h). Immunoblot analysis was conducted in one-way ANOVA with Dunnett’s multiple comparisons test on n = 3 biologically independent replicates. All the data are shown as mean ± SD and *p* values are presented in each individual *panel*. BN-PAGE, blue native-polyacrylamide gel electrophoresis; Co-IP, coimmunoprecipitation; CS, citrate synthase; ETC, electron transport chain; FCCP, carbonyl cyanide-p-trifluoromethoxyphenylhydrazone; Fe-S, iron-sulfur; MD, molecular dynamics; Ndufs1, NADH dehydrogenase Fe-S protein 1; ROS, reactive oxygen species.

We speculated that a prospective ETC redox hub would be highly prevalent in cell types that have a high demand for ATP but also primarily utilize oxidative phosphorylation as part of their metabolism. Therefore, we focused our discovery approach on H1299 metastatic lung cancer cells, a cell type that likely needs an efficient ETC battery, since they may require large amounts of ATP for several metastatic processes (14, 15), and directly compared them to A549 lung cancer cells (considered a less metastatic cell line), which would mainly utilize anaerobic glycolysis for their metabolic and proliferative needs (29). We first confirmed that H1299 cells had increased metastatic features compared to A549 cells and found an increase in migration, along with the levels of the metastatic marker SLUG and the expected decrease in E-cadherin (Fig. S1, *A* and *B*). Furthermore, we found that mitochondrial membrane potential (ΔΨm, energy capacity), mtROS (electron leak to superoxide) and oxygen consumption rate (OCR, surrogate for low-energy electrons reducing oxygen to water) were significantly elevated in H1299 metastatic cells, compared to A549 cells (Fig. 1, *A*–*C* and Fig. S2*A*), suggestive of a highly functioning ETC (3, 11). To ensure these mitochondrial differences were not simply due to variations in mitochondrial quantity, we measured mitochondrial mass using Nonyl Acridine Orange (NAO), which colocalized with tetramethylrhodamine methyl ester (TMRM) and observed lower levels in H1299 than A549 cells (Fig. S2, *B*–*D*). These findings suggest that the differences in ΔΨm, mtROS, and OCR observed between H1299 and A549 cells were most likely due to differences in mitochondrial function and not mitochondrial number/mass.

We hypothesized the increase in mtROS from H1299 metastatic cells may directly facilitate the transformation of individual ETC components into the ETC respirasome supercomplex (*via* oxidation of specific/candidate cysteine redox switches), and this could explain to some degree the increased ΔΨm and the directly related OCR (11). Therefore, we measured ETC supercomplex levels in H1299 and A549 cells and directly compared them to nonfailing human heart tissues, which are known to have abundant respirasomes (30, 31). We observed higher levels of respirasomes (CIn + IIIn + IVn) in human heart tissues and H1299 metastatic cells, than A549 cells, after loading the same amount of mitochondrial extracts resolved with digitonin (Figs. 1*D* and S3, *A* and *B*).

To understand if cysteine redox switches could be implicated in complex I incorporation into ETC supercomplexes, we focused our approach on Ndufs1 for several reasons. First, Ndufs1 is the largest component of complex I, second, it is assembled in a strategic position within complex I (see Movie 1) that if modified (*i*.*e*., Cys oxidation) could change its structural dynamics, and third, it has previously been implicated in ETC supercomplex formation (21). Therefore, to provide evidence that Ndufs1 could be important in our proposed model, we first performed coimmunoprecipitation (co-IP) against cysteine sulfonation posttranslational modification(s) from denatured mitochondrial extracts and confirmed cysteine sulfonation posttranslational modification on the Ndufs1 protein of metastatic H1299 cells (Fig. 1*E*). Then we performed co-IP against Ndufs1 from denatured mitochondrial extracts from A549 cells and metastatic H1299 cells and found that Ndufs1 was more oxidized in metastatic H1299 cells, compared to A549 cells (Fig. 1*F*), supporting our initial notion. To confirm that Ndufs1 is important for complex I function or supercomplex assembly, we generated a Ndufs1 KO A549 cell line, and found that (as expected) complex I stability, and subsequently ETC supercomplexes were completely disrupted, compared to parental cells (Figs. 1*G* and S4, *A* and *B*). In keeping, Ndufs1 KO cells had higher levels of mtROS (Fig. S4*C*) and significant nuclear DNA damage (Fig. S4*D*), providing evidence for its critical importance in complex I (and ETC) function. Based on these preliminary findings, we decided to move forward to understand if we could identify a potential redox hub within Ndufs1, which we could then artificially (by incorporating an oxidized Cys mimetic mutation) or physiologically (by increasing mtROS levels) oxidize in A549 cancer cells, to support the concept for a potential redox hub/sensor that could facilitate ETC respirasome formation.

To identify potential Cys redox switches within Ndufs1 that may form a redox hub, we mutated all known cysteine (C) residues previously shown to be oxidized *in vivo* (as stated in the Oximouse compendium (8)) to aspartic acid (D), which mimics a permanent sulfinylation oxidative modification (32). We found that overexpression of several different oxidative Cys mimetic mutants in Ndufs1 increased the formation and levels of the ETC respirasome, compared to WT overexpression of Ndufs1 (Fig. 1*H*). These data suggest that the free complex I was incorporated into the respirasome, indicating that our approach to normalizing respirasome levels to complex I level in the same native-PAGE immunoblot, rather than separate SDS-PAGE blots, provided a more accurate representation of respirasome assembly. We specifically focused on the C64D, C75D, C78D, and C92D mutants as they resulted in the highest levels of ETC respirasomes in A549 cells (Fig. 1*H*). Structural modeling indicated that these four cysteine residues form a hub around the Fe-S cluster N1b (Fig. 1*I*), suggesting that this region could serve as a candidate redox hub. To assess if this putative redox hub can be oxidized naturally, we measured the oxidation status of these Cys residues in normal (from unused donor) human heart tissues. Notably, neither the specific Cys residues nor the type of oxidative modifications appeared to influence ETC respirasome assembly (Fig. S4*E*). Given that the N1b cluster is unlikely to be oxidized by physiological levels of H_2_O_2_ (33), our results suggest that C64, C75, C78, and C92 may be the most critical components of the redox hub responsible for promoting ETC respirasome assembly.

To explore if oxidation of Ndufs1 is sufficient to promote respirasome formation, we employed a physiologically relevant approach using carbonyl cyanide-p-trifluoromethoxyphenylhydrazone (FCCP), an ionophore known to uncouple the ΔΨm from ATP synthase (similar to uncoupling proteins). FCCP has been shown to increase NADH oxidation to NAD^+^, enhance overall respiration, and promote cancer cell proliferation (34) (partially summarized in Fig. S5*A*). In line with these observations, we found that FCCP treatment of A549 cancer cells reduced the ΔΨm and the NADH/NAD^+^ ratio (Fig. S5, *B* and *C*). Additionally, mtROS levels exhibited a mild increase between 1 and 4 h posttreatment, followed by a decrease from 8 to 24 h (Figs. 1*J* and S5*D*). We found that C92 of Ndufs1 was oxidized to sulfonation 8 h after FCCP treatment (Fig. 1*K*), a time point that coincided with an observed increase in respirasome levels and activity (Figs 1*L* and S5*E*). Collectively, these data suggest that C64, C75, C78, and C92 form a redox-sensitive hub within Ndufs1, where their oxidative modifications appear to promote ETC respirasome formation.

### Oxidation of the redox hub within Ndufs1 can increase the distance between Fe-S clusters N5 and N6a of complex I, and this is reversed after formation of the respirasome supercomplex

**Figure 2:**
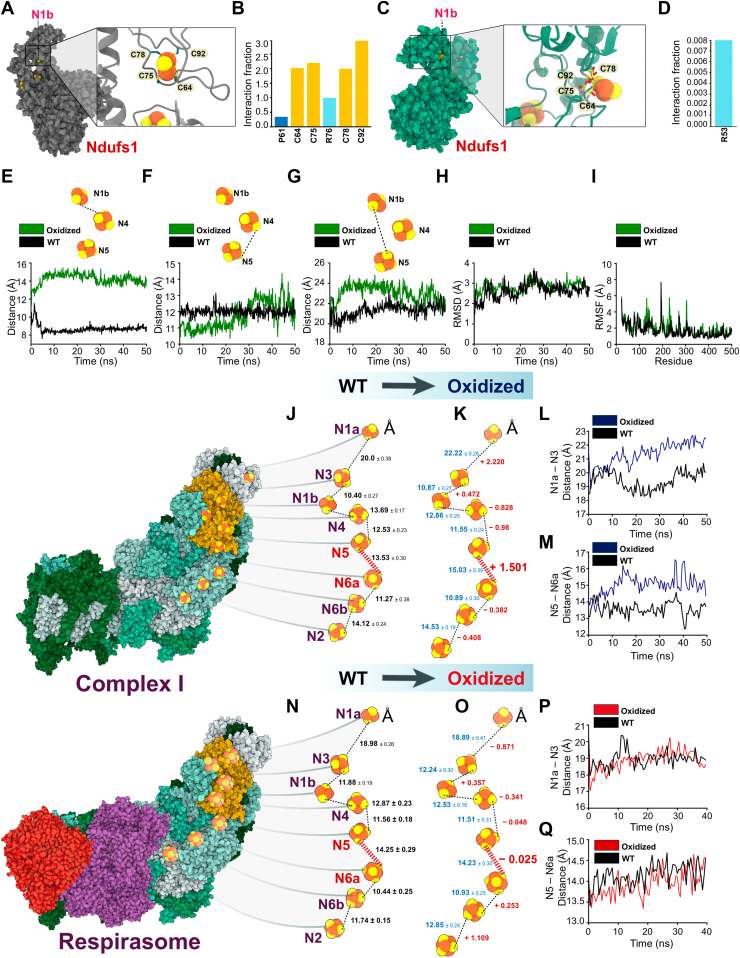
**Structural comparative analysis of the nonoxidized and oxidized redox hub within Ndufs1**. *A*, representation of cysteine residue orientations within the native Ndufs1 structure in relation to the N1b (FE-SFE-S cluster) following MD simulation. *B*, quantitative assessment of cysteine contact frequencies within native Ndufs1 over the course of the MD simulation. *C*, visualization of cysteine residue orientations within oxidized Ndufs1 following MD simulation. *D*, fractional analysis depicting contact frequencies between cysteine residues within oxidized Ndufs1. *E–G*, time-resolved measurement of distances between N1b, N4, and N5 atoms during the MD simulation. *H*, comparative analysis of backbone root mean square deviation for oxidized and WT ndufs1 across the MD simulation. *I*, comparative root mean square fluctuation of side chain atoms for oxidized *versus* WT Ndufs1. *J*, analysis of interatomic spacing within the complex I Fe-S cluster featuring WT Ndufs1during the last 5 ns of MD simulation. *K*, distance metrics between the complex I Fe-S cluster with oxidized cysteine Ndufs1 on the *left*, juxtaposed with the distance deviation from WT to oxidized forms on the *right*, highlighted in *red*. *L* and *M*, time-course assessment of spacing between n1a, N3, and N5, and N6a throughout the MD simulation. *N*, spacing analysis within the respirasome's Fe-S cluster with WT Ndufs1 during the last 5 ns of MD simulation. *O*, measurement of distances within the respirasome's Fe-S clusters with Ndufs1 containing oxidized cysteines on the *left*, and the comparative distance delta from WT to oxidized forms on the *right*, in *red*. *P* and *Q*, dynamic assessment of distances between N1a, N3, and N5, and N6a across the simulation duration within the respirasome. Fe-S, iron-sulfur; MD, molecular dynamics; Ndufs1, NADH dehydrogenase Fe-S protein 1.

To determine how our proposed redox hub could facilitate the formation of the ETC respirasome, we utilized an *in silico* structural modeling approach at atomic level resolution. We first assessed Ndufs1 in its isolated and nonoxidized native conformation (independent of other complex I proteins and cofactors). We found the four cysteine residues (C64, C72, C75, and C92) within the redox hub form coordination bonds with the N1b Fe-S cluster, which is augmented by noncovalent interactions with arginine (R)-53 and proline (P)-38 (Fig. 2, *A* and *B* and Fig. S6*A*). Upon Cys oxidation (to any of the four Cys residues), the interactions with the N1b Fe-S cluster are disrupted (Fig. 2, *C* and *D* and Movie 2 and Fig. S6*B*), resulting in a slight increase in distance between the N1a and N4 Fe-S clusters (Fig. 2*E*), while the interatomic separations between other Fe-S clusters, including N4 and N5 or N5 and N1a remained constant (Fig. 2, *F* and *G*). Quantitative structural analysis indicated negligible differences in the overall tertiary structure of Ndufs1 (Figs. 2*H* and S6*C*), but some significant (albeit minor) alterations suggestive of localized flexibility and dynamic protein behavior were noted (Fig. 2*I*). In addition, we found pronounced differences in the interaction landscape from amino acids 32 to 230 (Fig. S6, *D* and *E*). Overall, these data suggest that oxidation of the redox hub (in this setting) may facilitate only minor structural changes to Ndufs1, when in its isolated form.

To provide a more comprehensive and biological assessment, we next repeated our *in silico* modeling and analysis with Ndufs1 fully incorporated into complex I and in the presence of all associated cofactors. In this setting, when the redox hub was oxidized, a significant increase in the spatial distance of Fe-S clusters N1a-N3 and N5-N6a was observed (Figs. 2, *J*–*M* and Fig. S6, *F*–*J*). We focused our mechanistic approach on N5 (located in Ndufs1) and N6a (located in Ndufb8) as this intercluster distance exceeded 15 Å, well above the maximum distance needed for efficient electron flow (35), which alludes to a potential decrease in complex I activity. Because a decrease in complex I activity can be sensed by complex III, initiating the formation of the respirasome (36, 37, 38, 39, 40), we lastly assessed if integration into the respirasome could rescue the increased intercluster distance between N5 and N6a. We engineered an *in silico* model of the respirasome in the presence/absence of our oxidized redox hub and found that the formation of the respirasome could completely rescue the longer Angstrom distance (>15) between the N5 and N6a Fe-S clusters, restoring them to distances observed in the unmodified native state (Figs. 2, *N*–*Q* and Movie 3 and Fig. S6, *K*–*O*). Intriguingly, we noticed the N6a-N6b-N2 Fe-S clusters were an exception, where these clusters appeared more linear to each other (Fig. 2*O*), and we speculate that this could be a strategic shift in Fe-S clusters that would improve overall ETC efficiency. In summary, our structural modeling data suggest that oxidation of the redox hub will first decrease complex I activity, followed by its integration with complex III_2_ and IV to form the ETC respirasome and restore electron flow.

### Oxidation of the redox hub within Ndufs1 can decrease complex I activity, which then signals the recruitment and integration of complexes III_2_ and IV into the respirasome supercomplex to re-establish complex I activity

**Figure 3:**
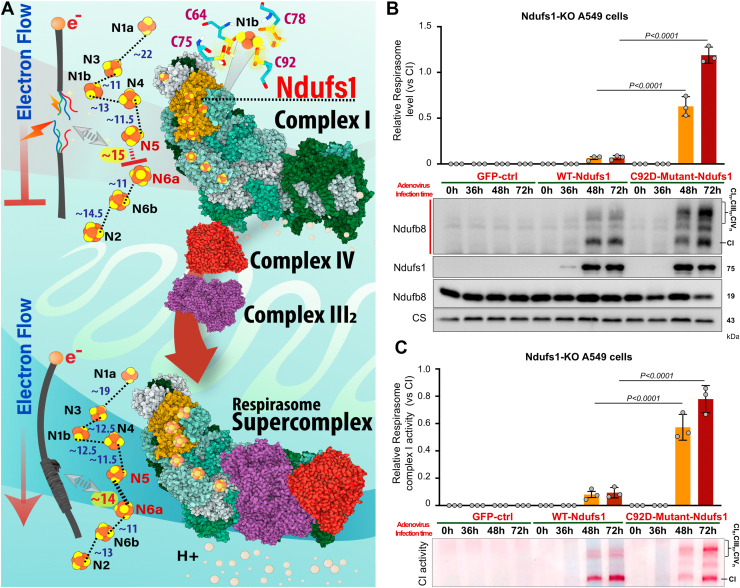
**Oxidation of the redox hub within Ndufs1 can decrease complex I electron flow and activity prior to formation of the ETC respirasome and subsequent reactivation of complex I**. *A*, schematic illustration of the assembly process of the respirasome supercomplex following oxidative modifications of Ndufs1 (see discussion section for detailed explanation). *B*, BN-PAGE and SDS-PAGE separation of mitochondrial proteins from A549 Ndufs1-KO cells, postinfection with GFP, WT Ndufs1, or C92D Ndufs1 mutant (Cysteine 92 mutated to aspartic acid, C92D) adenovirus (0–72 h). Immunoblot analysis employed antibodies against Ndufb8 for complex I and the respirasome supercomplex in native samples, and Ndufb8, Ndufs1, and citrate synthase (CS) in denatured samples. Student’s *t* test was used for comparison between WT and C92D-mutant groups, n = 3 biologically independent replicates. *C*, in-gel assay of mitochondrial complex I activity, demonstrating the restoration of NADH oxidase activity in complex I and increased activity in the respirasome supercomplex of Ndufs1-KO cells by infected with GFP, WT Ndufs1, or C92D Ndufs1 mutant (Cysteine 92 mutated to aspartic acid, C92D) adenovirus (0–72h). Data presented as the ratios of NADH oxidase activities between the respirasome supercomplex and complex I. All data are shown as mean ± SD and *p* values are presented in each *panel*. BN-PAGE, *blue* native-polyacrylamide gel electrophoresis; ETC, electron transport chain; Ndufs1, NADH dehydrogenase Fe-S protein 1.

Our structural findings indicate that oxidation of the redox hub within Ndufs1 would diminish complex I activity, which may in turn signal the recruitment and integration of complexes III_2_ and IV into the respirasome, to restore complex I function (Fig. 3*A*). To provide biological evidence for our theoretical *in silico* mechanism, we expressed either WT or C92D mutant Ndufs1 *via* adenovirus in a time-dependent manner in Ndufs1-KO A549 cells, and then measured both ETC respirasome levels and complex I activity (in both its independent state and when integrated into the respirasome). We found that re-expression of WT Ndufs1 increased free complex I levels and activity at 48 and 72 h post-adenovirus infection, compared to AdGFP controls (Fig. 3, *B* and *C*), while compared to WT Ndufs1, expression of C92D mutant Ndufs1 significantly increased respirasome complex I levels and activity at 48 and 72 h (Fig. 3, *B* and *C*). Overall, these findings support our structural modeling data and provide evidence for the mechanism that oxidation of the redox hub may first decrease complex I activity, prior to formation of the ETC respirasome and reactivation of complex I.

### C92D mutant Ndufs1 knock-in mitochondria have increased levels of the respirasome supercomplex and complex I activity and their functions are highly dependent on complex III_2_

**Figure 4:**
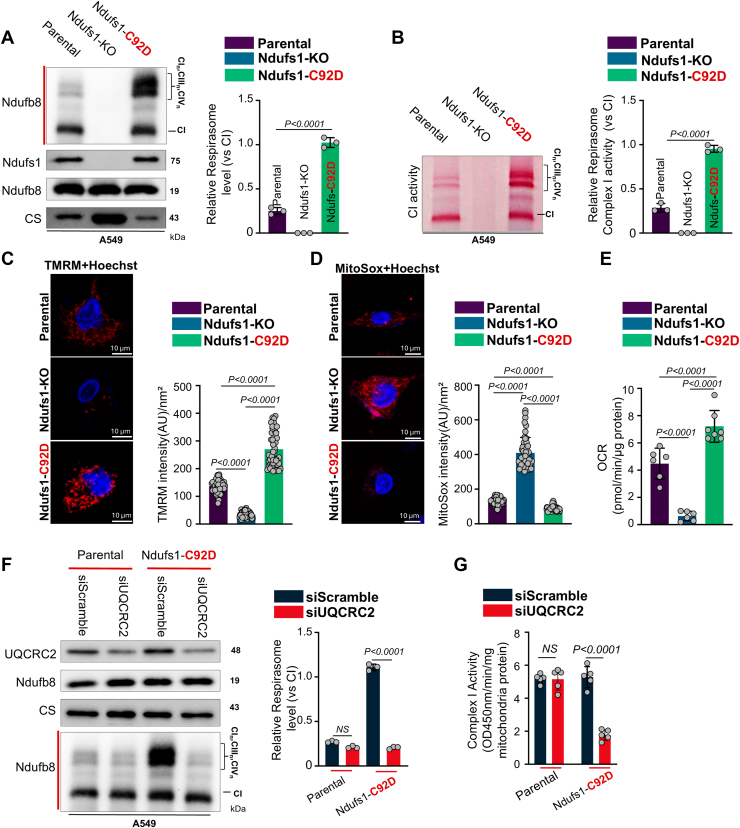
**C92D mutant Ndufs1 knockin mitochondria have increased levels of the respirasome supercomplex, where complex I activity and function are highly dependent on complex III**. *A*, BN-PAGE and SDS-PAGE separation of mitochondrial proteins from human A549 cells (parental, Ndufs1 KO, and C92D-Ndufs1 knockin), followed by immunoblot analysis using antibodies against Ndufb8 for complex I and respirasome supercomplex in native samples and Ndufb8, Ndufs1, and citrate synthase (CS) in denatured samples. One-way ANOVA with Dunnett’s multiple comparisons test on n = 3 biologically independent replicates. *B*, in-gel mitochondrial complex I activity was measured to determine the NADH oxidase activity ratio of the respirasome supercomplex to complex I. One-way ANOVA with Dunnett’s multiple comparisons test on n = 3 biologically independent replicates. *C*, mitochondrial membrane potential (ΔΨm) assessment in A549 cells (parental, Ndufs1-KO, and C92D-Ndufs1 knock-in) using tetramethylrhodamine methyl ester (TMRM). Confocal images illustrate increased ΔΨm in C92D-Ndufs1 mutant cells and decreased ΔΨm in Ndufs1-KO cells compared to parental cells. One-way ANOVA with Tukey’s multiple comparisons test on n = 50 cells/group. AU, arbitrary unit. *D*, mitochondrial ROS were analyzed in A549 cells (parental, Ndufs1-KO and C92D-Ndufs1 knock-in) labeled with MitoSox. Representative confocal images show lower mitochondrial ROS production in C92D-Ndufs1 mutant cells and higher mitochondrial ROS production in Ndufs1-KO cells than parental cells. One-way ANOVA with Tukey’s multiple comparisons test on n = 50 cells/group. AU, arbitrary unit. *E*, analysis of oxygen consumption rate (OCR) in A549 cells (parental, Ndufs1-KO and C92D-Ndufs1 knock-in) were assessed by Seahorse assay, showing higher basal OCR level in C92D-Ndufs1 mutant knock-in cells and lower basal OCR level in Ndufs1-KO cells than parental cells. One-way ANOVA with Tukey’s multiple comparisons test on n = 6 to 7 wells/group. *F*, BN-PAGE and SDS-PAGE separation of mitochondrial proteins from parental A549 cells or cells transfected with UQCRC2 siRNA or scramble siRNA as control, followed by immunoblot analysis using antibodies against Ndufb8 for complex I and respirasome supercomplex in native samples and UQCRC2, Ndufb8, and citrate synthase (CS) in denatured samples. One-way ANOVA with Dunnett’s multiple comparisons test on n = 3 biologically independent replicates. Statistical significance was calculated with two-way ANOVA, followed by Sidak’s multiple comparison. *p* values are presented in each *panel*. NS, nonsignificant. *G*, total NADH-dehydrogenase activity assays of CI in parental A549 cells and C92D-Ndufs1 mutant knock-in cells transfected with UQCRC2 siRNA or scramble siRNA as control. Data are presented as mean values ± S.D of n = 6 biological replicas. Statistical significance was calculated with two-way ANOVA followed by Sidak’s multiple comparison. *p* values are presented in each *panel*. BN-PAGE, blue native-polyacrylamide gel electrophoresis; Ndufs1, NADH dehydrogenase Fe-S protein 1; NS, nonsignificant.

We speculated that the respirasome levels and activity from C92D mutant Ndufs1 would be highly dependent on complex III_2_ (which migrates to complex I when its activity is decreased (36, 37, 38, 39)). To test this, we generated a partial C92D knock-in mutant Ndufs1 cell line, which had increased respirasome levels and activity, compared to parental or Ndufs1 KO A549 cells (Figs. 4, *A* and *B*, Fig. S7, *A* and *B* and Fig. S8, *A* and *B*). Furthermore, ΔΨm was significantly elevated (Figs. 4*C* and S8*C*), while mtROS was decreased (Fig. 4*D*, Figs. S8*D* and S9) and OCR was increased (Figs. 4*E* and S10) in C92D Ndufs1 knock-in cells, compared to parental or Ndufs1 KO cells.

To assess if the increased respirasomes from the C92D mutant Ndufs1 mitochondria have more efficient electron flow, compared to either Ndufs1 KO or parental mitochondria, we integrated mitochondrial membranes onto a biobattery platform (to measure direct electrical current), but we first determined the appropriate biological substrates required for electron donation in this *ex situ* system. The pyruvate dehydrogenase complex has previously been shown to interact with the ETC respirasome (41, 42), and we confirmed this interaction with both WT and C92D mutant Ndufs1 (Fig. S11*A*). These data suggest that pyruvate and NAD^+^ could be viable substrates to generate (*via* pyruvate dehydrogenase complex) spatially localized NADH that can then directly donate its electrons to complex I of the respirasome. This approach is more desirable than providing NADH extracellularly, since it is highly prone to degradation (43). Next, we isolated and immoblized mitochondrial membranes onto a carbon paper electrode (Fig. S1*B*), and observed significantly more current (in the presence of pyruvate and NAD^+^) from C92D Ndufs1 knock-in, compared to parental or Ndufs1 KO samples (Fig. S11, *C* and *D*). These data confirm that C92D Ndufs1 knock-in mitochondria have more efficient electron flow than parental or Ndufs1 KO mitochondria.

To assess if complex III_2_ is indeed important for stabilizing complex I activity in the respirasomes of the C92D mutant Ndufs1 mitochondria, we inhibited complex III_2_ function by decreasing the levels of UQCRC2 (a large complex III protein). Knockdown of UQCRC2 (with siRNA encoding multiple targets within UQCRC2) not only decreased complex I levels and activity within the respirasome (Fig. 4*F*, Figs. S12 and S13) but also total complex I activity in C92D knock-in cells, but not parental A549 cells, when compared to scrambled siRNA-treated cells (Fig. 4*G*). These data suggest that the respirasome within the C92D mutant Ndufs1 mitochondria have a high dependence on complex III_2_ function for stable respirasome levels and activity and are in keeping with the concept that the recruitment of CIII_2_ to CI (and formation of the respirasome) is sufficient to rescue the decreased CI activity (36, 37, 38, 39).

### Disruption of the ETC supercomplexes with MitoTam can increase the therapeutic efficacy of reactive oxygen species (ROS)-inducing chemotherapeutics in C92D mutant Ndufs1 knock-in A549 cells or metastatic H1299 lung cancer cells, compared to parental A549 cancer cells

**Figure 5:**
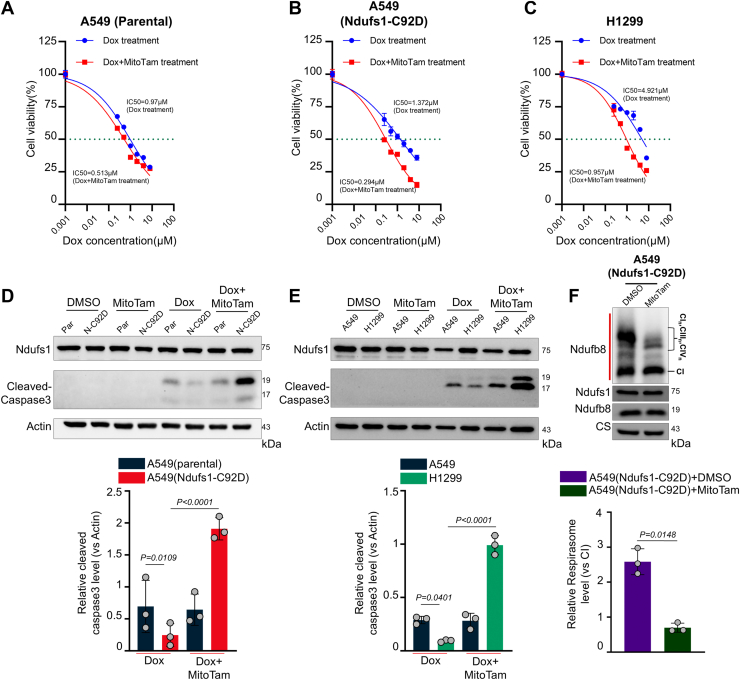
**C92D mutant Ndufs1 knock-in A549 cells (N-C92D) and metastatic H1299 cells are more resistant to chemotherapy (doxorubicin)-mediated cell death, compared to parental A549 cells (Par)**. Dose-response curves showing the viability of parental A549 cells *A*, C92D mutant Ndufs1 knock-in A549 cells *B*, and metastatic H1299 cells *C*, treated with increasing concentrations of doxorubicin with or without MitoTam (200 nM) pretreatment (12 h) for 24 h. Comparison of IC50 values for doxorubicin treatment among the three cell lines, highlighting increased resistance in C92D mutant Ndufs1 knock-in A549 cells and metastatic H1299 cells. Data are presented as mean ± SD of three independent experiments. *D*, representative Western blot analysis of cleaved caspase-3 expression in parental A549 cells and C92D mutant Ndufs1 knock-in A549 cells after dimethyl sulfoxide (DMSO), MitoTam (200 nM), doxorubicin (1 μM) or doxorubicin + MitoTam treatment, with actin as a loading control. Data are presented as mean ± SD of three independent experiments. Statistical analysis was performed using two-way ANOVA, followed by Sidak’s multiple comparisons. *p* values are presented in each *panel*. *E*, representative Western blot analysis of cleaved caspase-3 expression in parental A549 cells and metastatic H1299 cells after DMSO, MitoTam (200 nM), doxorubicin (1 μM), or doxorubicin + MitoTam treatment, with actin as a loading control. Data are presented as mean ± SD of three independent experiments. Statistical analysis was performed using two-way ANOVA, followed by Sidak’s multiple comparisons. *p* values are presented in each panel. *F*, BN-PAGE and SDS-PAGE separation of mitochondrial proteins from C92D mutant Ndufs1 knock-in A549 cells treated with DMSO or MitoTam (200 nM) for 12 h. Immunoblot analysis employed antibodies against Ndufb8 for complex I and the respirasome supercomplex in native samples, and Ndufb8, Ndufs1, and citrate synthase (CS) in denatured samples. Student’s *t* test was used for comparison between two groups, n = 3 biologically independent replicates. BN-PAGE, blue native-polyacrylamide gel electrophoresis; Ndufs1, NADH dehydrogenase Fe-S protein 1.

To provide therapeutic significance to our findings, we assessed our mechanism for increasing the therapeutic efficacy of standard and clinically used ROS-inducing chemotherapeutics. We utilized the anthracycline doxorubicin or the alkylating agent cisplatin, two common chemotherapeutics for lung cancer, which can increase mtROS levels, promoting nuclear DNA damage and cell death. IC50 values revealed that C92D mutant Ndufs1 knock-in A549 cells and metastatic H1299 cells exhibited significantly higher resistance to doxorubicin and cisplatin, than parental A549 cells (Fig. 5, *A*–*C*). This was further supported by cleaved caspase-3 analysis, where both doxorubicin and cisplatin treatments induced lower levels of cleaved caspase-3 in C92D mutant Ndufs1 knock-in A549 cells and metastatic H1299 cells, than parental A549 cells, suggesting less cell death (Fig. 5, *D* and *E*). We speculated that although the increase in ROS by doxorubicin or cisplatin would facilitate cell death in the majority of A549 cancer cells, it could potentially increase Ndufs1 oxidation, and subsequently the formation of ETC supercomplexes in a small subset of cells, facilitating chemoresistance. Indeed, we found that treatment with either doxorubicin or cisplatin could increase both, the cysteine sulfonation modification on Ndufs1 (Fig. S14, *A* and *B*), but also mitochondrial ETC supercomplex formation (Fig. S14*C*) in the surviving A549 cells, providing a potential mechanism for ROS-mediated chemoresistance (at least in the case of doxorubicin or cisplatin).

To provide a therapeutic approach for doxorubicin or cisplatin-resistant lung cancer cells, we utilized MitoTam, a small molecule compound that can initiate ETC supercomplex disassembly (44) and was reported as a cytostatic drug that can reduce oxidative phosphorylation, which is currently being tested in an early phase clinical trial (45). We found that MitoTam can effectively decrease ETC supercomplex levels in C92D mutant Ndufs1 knock-in cells (Fig. 5*F*). MitoTam pretreatment reduced the IC50 of doxorubicin in C92D mutant Ndufs1 knock-in A549 cells and metastatic H1299 cells (Fig. 5, *A*–*C*), while also increasing cleaved caspase-3 levels to a similar degree as doxorubicin- or cisplatin-treated parental A549 cells (Fig. 5, *D* and *E* and Fig. S15, *A* and *B*). Overall, these data suggest that ETC supercomplex disassembly could be a therapeutic option to overcome resistance or improve therapeutic efficacy for chemotherapeutics designed to increase cellular ROS levels.

## Discussion

Here, we report the identification of a redox hub within Ndufs1 of the mitochondrial ETC, that when oxidized, can initiate the formation of the ETC respirasome. Mechanistically, we show that once oxidized, the redox hub can temporarily decrease complex I activity, prior to integration with complex III_2_ and IV to form the ETC respirasome, which subsequently re-establishes forward electron flow and complex I activity (see mechanism in Fig. 3*A*). Several studies have reported that a decrease in complex I activity can be sensed by complex III_2_, which can then migrate toward complex I to initiate the formation of different ETC supercomplexes (36, 37, 38, 39, 40). Taken together with our molecular data, we speculate that the initial decrease in complex I activity (when the redox hub is oxidized) is most likely sensed by complex III_2_, since complex I electrons eventually enter the ubiquinone pool, resulting in the migration of a complex III_2_/IV unit toward complex I to improve/rescue electron flow, by forming the ETC respirasome (Fig. 3*A*).

A limitation of our study is that we did not address the relative importance for the oxidation of each cysteine residue within our redox hub in facilitating the formation of the respirasome supercomplex. Our work does appear to suggest that C92 is the most receptive to oxidation, within our proposed redox hub, while C64 did not appear to be oxidized in any of our investigated samples. Our structural modeling seems to suggest that the microenvironment around C92 is receptive toward oxidation, while C64 is likely to be buried (or less accessible, Fig. S16), which could provide some explanation for our biological findings.

A further limitation of this study is that it did not explore whether other potential cysteine redox switches either within Ndufs1 or in other ETC complexes may promote respirasome formation. For example, we found that C554, C564, and C727 of Ndufs1 could all form higher levels of respirasomes when mutated to aspartic acid (Fig. 1*H*). Future studies should investigate the significance/purpose for these ETC redox switches, and specifically if any of them can also promote respirasome formation. Most likely, multiple redox sensors/switches are present within the ETC to allow for the quick and efficient transformation to the ETC respirasome in response to pathophysiological levels of cellular or mitochondrial ROS. Finally, future work should assess the role of these redox-derived ETC supercomplexes in relation to other conditions, like cardiotoxicity or other types of heart failure, where its disruption could result in a less efficient ETC and perhaps compromised ATP synthesis.

This study provides a potential therapeutic platform for improving chemotherapy efficacy. Because ETC respirasomes allow for tolerance against chemotherapeutics, specifically those that aim to increase overall ROS levels, our study suggests that drugs that can disrupt ETC respirasomes could provide a new therapeutic platform against chemoresistance, as in our example for doxorubicin or cisplatin chemoresistance in C92D mutant Ndufs1 knock-in A549 cells or H1299 metastatic lung cancer cells. Future studies should evaluate if increased ETC respirasomes can provide other advantages for cancer cells. For example, it may be plausible that increased ETC respirasomes and improved mitochondrial function (in cancer cells) can directly facilitate metastatic transformation or changes in cell fate (*via* mitochondrial signaling to the nucleus).

Finally, our findings suggest that integration of ETC respirasomes as part of a biofuel cell battery could be a novel technological option. The implications of these findings need be further developed, but we envision this type of technology could be used to power nanodevices where conventional batteries would be harmful. For example, smart contact lenses are attractive devices that could have significant value for health, military, or daily life purposes (46, 47). Because tear fluid has all the necessary substrates, including pyruvate and NAD^+^ (48) for an ETC respirasome-derived biofuel cell, it could provide a safe and effective biobattery for this type of technology, but could be extended to other types of wearable or integrated biological nanodevices, once optimized.

## Experimental procedures

The study protocol for acquisition of human nonfailing heart tissue samples conformed to the ethical guidelines of the 1975 Declaration of Helsinki and was reviewed by the ethics review board at the University of Alberta (Pro00035875).

### A549 cell and H1299 cell culture

A549 human lung carcinoma epithelial cells were purchased from American Type Culture Collection and cultured in Dulbecco's modified Eagle's medium (DMEM) with 10% fetal bovine serum (FBS, Sigma) and 1% penicillin-streptomycin (Gibco) at 37 °C in a humidified incubator containing 9% CO_2_. The C92D Ndufs1 knock-in A549 cell lines, along with the Ndufs1 KO A549 cell lines were generated from GenScript and the sequencing and validation for these cell lines are provided in the supplementary information section. H1299 human non–small cell lung carcinoma cells were purchased from American Type Culture Collection and cultured in RPMI-1640 supplemented with 10% FBS (Sigma) and 1% penicillin-streptomycin (Gibco) at 37 °C in a humidified incubator containing 9% CO_2_. Prior to live-cell imaging or other comparative experiments between A549 and H1299, the media for H2199 cells was switched to the same culture media as the A549 cells, to maintain similar culture conditions during the experiment, which would allow for direct comparisons between the two cell lines.

### *Mycoplasma* detection by PCR assay

Cell lines were cultured without mycoplasma-active antibiotics until 90 to 100% confluence. For supernatant testing, 100 μl of culture medium was transferred to a sterile tube, boiled at 95 °C for 5 min, and briefly centrifuged to pellet debris. The supernatant was used directly as template. PCR reactions (25 μl total volume) contained 23 μl prepared DNA polymerase/rehydration buffer (JumpStart Taq, 0.5 μl/reaction), 2 μl boiled supernatant (or nuclease-free water for negative control), and positive controls used kit-provided *mycoplasma* DNA (25 μl reaction). Cycling conditions: initial denaturation: 94 °C, 2 min; 40 cycles: 94 °C/30s, 55 °C/30 s, 72 °C/40s; final hold: 4 to 8 °C. PCR products (8 μl/lane) were resolved on a 1.2% agarose gel (100 V, 25 min). Internal control (481 bp) confirmed valid PCR; mycoplasma-positive samples showed a ∼260 bp band. All tested supernatants were negative and are provided in the supplementary information section.

### Isolation of mitochondria

Mitochondria were isolated from A549 cells using differential centrifugation according to the manufacturer’s instructions (#89874, Thermo Fisher Scientific). Briefly, the pellets of 2 × 10^7^ A549 cells were harvested in a 2 ml microcentrifuge tube. The cell pellets were then placed on ice and 800 μl of reagent A was added to suspend the pellets by a 5s-vortex at medium speed. The resulting cell suspension was then transferred to a precooled manual glass-Teflon homogenizer for homogenization by 50 strokes on ice, following which the cell lysate was transferred to a new tube. To recover any remaining lysate, 200 μl of reagent A was used to rinse the homogenizer, and the rinse was added to the tube. Next, 1000 μl of reagent B was added to the tube, which was inverted several times to mix the lysate. The mixture was centrifuged at 700 × *g* for 10 min at 4 °C to pellet the cell debris and nuclei. The resulting supernatant, containing mitochondria, was transferred to a new tube. The mitochondria were pelleted by centrifugation at 12000 × *g* for 15 min at 4 °C. The pellet was washed with 500 μl of reagent C, and this washing step was repeated once more. The final mitochondrial pellet was resuspended in STE buffer (0.32 M sucrose, 1 mM EDTA, and 10 mM Tris–HCl, pH 7.4). The protein concentration of the mitochondrial suspension was determined using the bicinchoninic acid (BCA) Protein Assay Kit (Thermo Fisher Scientific).

### Preparation of mitochondrial protein lysis

Mitochondria were pelleted by centrifugation at 14,000 × *g* for 15 min at 4 °C. The pellet was resuspended in 100 μl of aminocaproic acid buffer (1.5 M aminocaproic acid, 50 mM Bis-Tris, pH 7) containing protease inhibitors. Resolution buffer (with a digitonin/protein ratio of 10 g/g, supplemented with aminocaproic acid buffer to a final volume of 200 μl) was added to the samples, which were then incubated on ice for 20 to 30 min. The supernatant was collected by centrifugation at 16,000 × *g* for 15 min at 4 °C. For blue native-polyacrylamide gel electrophoresis, 10× Native Loading Buffer (composed of 5% [w/v] Coomassie Brilliant Blue G, 750 mM aminocaproic acid, 50 mM Bis-Tris [pH 7], 0.5 mM EDTA [pH 8], and 50% [v/v] glycerol) was added to the lysis buffer, and 20 μl aliquots of each sample were prepared. Alternatively, for SDS-PAGE, samples were mixed with SDS loading buffer and boiled for 5 to 10 min. All samples were stored at −80 °C until further use, to avoid repeated freeze-thaw cycles.

### Blue native-polyacrylamide gel electrophoresis

Fifteen microliters of sample was loaded in each well of 3% to 12% Native Gel (Invitrogen) and electrophoresed using an XCell SureLock Mini-Cell (Invitrogen) at a constant voltage (50 V for 30 min and 250 V for 2 h) at 4 °C as previously described (49). Proteins were visualized using Coomassie R20 staining, Western blot analysis or in-gel catalytic activity staining for respirasome supercomplexes and isolated complexes. For Western blot analysis, the gel was immersed and denatured in Tris buffer (containing 1% SDS, 100 mM acetic acid, and 300 mM Tris) for 15 min at 37 °C.

### Immunoblot analysis

For blue native-polyacrylamide gel electrophoresis, proteins were transferred onto polyvinylidene difluoride membranes (Milipore, 0.45 μm) by wet transfer at a constant voltage (20–30 V) for 20 h at 4 °C. Polyvinylidene difluoride membranes were air-dried out completely after transfer and then destained with methanol until bands were barely visible. Next, the membranes were blocked with 5% nonfat milk blocking buffer for 4 h at room temperature (RT) or overnight at 4 °C. For SDS-PAGE, proteins were transferred onto nitrocellulose membrane (Millipore, 0.45 μm) by semidry transfer at a constant voltage (25 V) for 10 min and then blocked with 5% nonfat milk blocking buffer for 1 h at RT. After incubation with the primary and secondary antibody subsequently, immunodetection was performed using the ChemiDoc Imaging Systems (Bio-Rad) with standard techniques with Western ECL Substrate (Cytiva RPN2106, Sigma). Primary antibodies specific for Ndufs1 (1:1000; ab169540, Abcam), Cysteine sulfonate (1:1000; ab176487), Ndufb8 (1:1000; ab192878, Abcam), SDHB (1:1000; 92649S, Cell Signaling Technology), UQCRC2 (1:1000; 99258S, Cell Signaling Technology), citrate synthase (1:1000; 14309S, Cell Signaling Technology), COX IV (1:1000; 4844S, Cell Signaling Technology), phospho histone H2A.X (1:1000; 5438, Cell Signaling Technology), cleaved caspase-3 (1:1000; 9661, Cell Signaling Technology), actin (1:1000; ab179467, Abcam), PDH-E1α (1:200; sc-377092, Santa Cruz), E-cadherin (1:1000; 3195, Cell Signaling Technology), Slug (1:1000; 9585, Cell Signaling Technology) were used. Bands were quantified with ImageJ software (NIH; https://imagej.net/software/imagej/). All uncropped immunoblots, along with the molecular weight markers are provided in the supplementary information section.

### In-gel complex I activity assay

After electrophoretic separation, the gels were immersed directly in the complex I (CI) enzymatic activity solution (2 mM Tris–HCl pH7.4, 2.5 mg/ml iodonitrotetrazolium (Sigma), 0.1 mg/ml NADH (Sigma)) for 30 to 60 min at RT. Once the bands were clear, the gels were wrapped in transparent film paper and the signals captured using a scanner. Images were analyzed using ImageJ.

### Complex I activity kit (colorimetric)

All procedures were conducted following the manufacturer's instructions (ab109721). Briefly, cells were collected after transfected with siRNA for 72 h, following by protein extraction and concentration dilution to 50 μg/ml. Two hundred microlitersof sample is loaded per well. Microplate was incubated for 3 h at RT on a plate shaker. After adding the assay solution to the wells, plate was placed into the plate reader and CI activity was analyzed by measuring the absorbance (450 nm) in a kinetic mode at RT for up to 30 min.

### MitoSox and TMRM staining and confocal imaging

Accumulation of ROS and mitochondrial membrane potential were assessed separately using MitoSox and TMRM staining according to the manufacturer's protocols. Briefly, 1.5 × 10^5^ A549 cells were collected and seeded onto glass-bottom culture dishes 1 day prior to staining. The cells were washed twice with PBS and incubated in 2 ml of serum-free DMEM. TMRM, MitoSox, and Hoechst were added to the medium to a final concentration of 10 nM, 5 μM, and 5 μg/ml, respectively, and thoroughly mixed using a pipette. After a 30-min incubation period, the cells were washed twice with PBS and supplemented with 2 ml of complete medium. For the live imaging part, FCCP (1 μM) was added to the dishes, and live imaging was conducted under conditions of 9% CO_2_ at 37 °C for 5 min before and after FCCP treatment. Imaging was performed within 10 min per dish using confocal microscopy (Carl Zeiss 780 LSM).

### NAO staining

Briefly, 1.5 × 10^5^ A549 cells or H1299 cells were seeded onto glass-bottom culture dishes 1 day prior to staining. Prepare the NAO (Thermo Fisher Scientific) working solution (1 μM in PBS) fresh and protect it from light. The cells were washed twice gently with PBS and incubated in 2 ml of NAO working solution. After a 30-min incubation period, the cells were washed twice with PBS and fixed with 4% paraformaldehyde for 10 min at RT. The cells were washed twice gently with PBS and incubated with 4′,6-diamidino-2-phenylindole for 10 min at RT in the dark. Wash the cells 3 times with PBS to remove excess 4′,6-diamidino-2-phenylindole. Imaging was performed using confocal microscopy (Carl Zeiss 780 LSM).

### Comet assay

All procedures were conducted following the manufacturer's instructions (ab238544). Briefly, the agarose gel was heated until it liquefied, following which 76 ml of agarose was added per well onto the Comet slide to form a base layer. Cell samples were prepared by resuspending cells at a concentration of 1 × 10^5^ cells/ml in precooled PBS. The cell samples were then combined with Comet Agarose at a 1:10 ratio (v/v), thoroughly mixed, and immediately transferred at 75 μl per well onto the top of the base layer. After the agarose solidified, the slides were transferred to a container containing precooled lysis buffer and incubated for 60 min at 4 °C in the dark. Subsequently, the lysis buffer was replaced with precooled alkaline solution, and the slides were further incubated for 30 min at 4 °C in the dark. The slides were then transferred to an electrophoresis chamber, and the device was run at a constant voltage of 35 V for 25 min. Following electrophoresis, the slides were rinsed twice with precooled double distilled water and fixed with 70% ethanol for 5 min. After removal from the fixative, the slides were air-dried completely for at least 1 h. Subsequently, 100 μl per well of Vista Green dye was added and incubated for 15 min at RT. Images were acquired using confocal microscopy (Carl Zeiss 780 LSM), and the area of the tail was measured using ImageJ.

### Oxygen consumption rate

Real-time measurements of OCR were conducted using a Seahorse XF Analyzer (Agilent Technologies). A549 cells were seeded at a density of 2 × 10^4^ cells per well in 100 μl of media in XFp cell culture microplates and allowed to adhere overnight. Prior to the assay, cells were washed and incubated with Seahorse XF Base Medium, following the manufacturer's instructions. Seahorse Wave software (https://www.agilent.com/en/product/cell-analysis/real-time-cell-metabolic-analysis/xf-software/seahorse-wave-desktop-software-740897) was utilized for data analysis. Subsequently, after completion of the XF assay, cells were lysed with radio-immunoprecipitation assay buffer lysis buffer (30 μl per well), and the protein concentration was determined using the BCA protein assay. OCR data were normalized to protein concentration and expressed as picomoles per min per microgram of protein (pM/min/μg protein).

### Electron paramagnetic resonance spectroscopy measurement

Cells were seeded at a density of 1 × 10^6^ cells per well in a 6-well plate and allowed to adhere overnight. Subsequently, cells were trypsinized from the bottom of the plate and washed once with serum-free RPMI 1640 medium. The cells were then resuspended in 200 μl of carbogen-flushed serum-free RPMI 1640 medium. Five microliters of mito-tempo-H (1 mM) was then added to each well, resulting in a final concentration of 25 μM, followed by incubation at RT for 10 min. The cells were then transferred to Eppendorf tubes and placed on ice to slow the reaction. Three capillary tubes were loaded with each sample and sealed for detection of reactive oxygen species using electron paramagnetic resonance spectroscopy.

### Molecular modeling and dynamic simulations

The initial structure of NDUFS1, extracted from the active state of mouse mitochondrial complex I (PDB ID: 6G2J, chain G), was prepared for simulations using the Maestro Schrödinger suite's Protein Preparation Wizard. The preparation process entailed the addition of missing side chains facilitated by Epik, and the optimization of ionizable residues by pKa predictions using PROPKA (50). To refine the system, water molecules positioned beyond 5 Å from protein residues were eliminated. After this, the proteins were subject to a restrained minimization utilizing the OPLS4 force field (51). Molecular dynamics (MD) simulations were executed with Desmond software (https://www.schrodinger.com/platform/products/desmond/), adopting an orthorhombic box with a 10Å-boundary buffer, filled with the TIP3P water model, and ensuring charge neutrality to mimic physiological conditions. The simulations proceeded for 50 ns under the NPT ensemble to maintain a constant temperature of 300 K and pressure at 1.01325 bar, employing the OPLS4 force field throughout. Concurrently, a separate MD simulation of the entire complex I (PDB ID: 6G2J) was conducted over a similar 50 ns timeframe following the identical protocol described above. To construct the respirasome structure, the mouse complex I (PDB ID: 6G2J) was integrated with murine complexes III and IV (PDB IDs: 7O3H and 7O3C), emulating the architecture of the human respiratory supercomplex I1III2IV1 (5XTH). This reference model was chosen over the cryo-EM structure (PDB ID: 5XTH) due to the latter's suboptimal representation of coordination bonds around the Fe-S cluster. The assembled respirasome structure was then subjected to a 40 ns MD simulation, adhering to the specified conditions previously mentioned. The data from these simulations were analyzed using the Schrödinger Maestro suite and visual molecular dynamics software (52), with graphical representations generated through Mol∗ (Molstar (53) and ChimeraX (54)).

### Electrochemical assay

Five hundred microliters of mitochondrial sample from C92D Ndufs1 mutant knock-in, Ndufs1 KO, or parental cells were diluted in mitochondrial buffer to a final protein concentration of ∼0.5ug/ul. Nafion 117 in lower aliphatic alcohols (Sigma 70160), vortexed for 15 s, and 40 ul of this solution was drop-cast on 0.25 cm^2^ functional area, machine-cut carbon paper electrodes, and then dried under vacuum for 2 to 3 h. Cyclic voltammetry was performed using an Admiral Squidstat Prime using a standard three-electrode cell consisting of the carbon paper working electrode, a saturated calomel electrode reference, and a Pt mesh counter electrode. Scans were performed from 0.6 to −1 V *versus* saturated calomel electrode in 6 M NaNO_3_ at 25 °C and 30 mV/s in the presence and absence of 1 mM NAD^+^ and 100 mM sodium pyruvate. Data presented is the average of three separately prepared electrodes.

### Immunoprecipitation

Co-IPs were conducted using DynaGreen Protein A/G Magnetic Beads (80105G, Thermo Fisher Scientific) and Pierce immunoprecipitation (IP) lysis buffer (87787, Thermo Fisher Scientific), following the manufacturer’s instructions. Briefly, cells were pelleted and rinsed with prechilled PBS before being lysed in IP lysis buffer supplemented with IP buffer. Heart tissues were ground in a liquid nitrogen bath, and the resulting tissue powders were suspended in IP lysis buffer with IP buffer and sonicated. After a 30-min incubation at 4 °C on a rotator, the lysates were centrifuged to isolate the soluble fraction. Protein concentration was determined using a BCA kit. Next, 500 μl of IP lysis buffer containing 500 μg of protein was incubated overnight at 4 °C on a rotator with 10 to 12 μg of the specified antibodies. Magnetic beads were then added to the lysate and incubated at 4 °C on a rotator for 2 h. The beads containing the target proteins were washed and resuspended in 30 μl of cold lysis buffer and 30 μl of 2x Laemmli sample buffer (Bio-Rad), followed by boiling for 5 to 10 min. Ten microliters of the samples were analyzed by Western blotting, while the remaining samples were purified by gel electrophoresis for subsequent mass spectrometry analysis.

### Protein digestion for mass spectrometry

Samples underwent in-gel trypsin digestion. Briefly, the samples were first reduced (10 mM BME in 100 mM bicarbonate) and alkylated (55 mM iodoacetamide in 50 mM bicarbonate), followed by overnight digestion at 37 °C using Promega sequencing grade modified trypsin. Tryptic peptides were extracted from the gel using a solution of 97% water, 2% acetonitrile, and 1% formic acid, followed by a second extraction with a solution comprising 50% of the first extraction buffer and 50% acetonitrile. The combined supernatant peptides were then dried using a speed vacuum, desalted, and resuspended in a 0.1% formic acid aqueous solution containing 4% acetonitrile for nano-HPLC-MS/MS analysis. The proteomic data are provided in the supplementary information section.

### Nano-HPLC/MS/MS

Peptides were separated using a nanoflow LC (Easy-nLC 1000, Thermo Fisher Scientific) coupled to an Orbitrap Q Exactive mass spectrometer (Thermo Fisher Scientific) equipped with an EASY-Spray capillary HPLC column (ES902A, Thermo Fisher Scientific). The mass spectrometer operated in data-dependent acquisition mode with a resolution of 35,000 and *m/z* range of 300 to 1700. The twelve most intense multiply charged ions were sequentially fragmented using HCD dissociation with a normalized collision energy of 26, and spectra of their fragments were recorded in the Orbitrap at a resolution of 17,500. Precursors selected for dissociation were dynamically excluded for 30 s after fragmentation. Data were processed using Proteome Discoverer 2.4 (Thermo Fisher Scientific; https://www.thermofisher.com/us/en/home/industrial/mass-spectrometry/liquid-chromatography-mass-spectrometry-lc-ms/lc-ms-software/multi-omics-data-analysis/proteome-discoverer-software.html?erpType=Global_E1), and searches were conducted against the UniProt database (UP000005640 for human) using SEQUEST (Thermo Fisher Scientific). Search parameters included a strict false discovery rate of 0.01, a relaxed false discovery rate of 0.05, a precursor mass tolerance of 10 ppm, and a fragment mass tolerance of 0.01 Da. Peptides were searched with cysteine oxidation in the formation of sulfenic acid, sulfinic acid, and sulfonic acid and oxidized methionine, and deamidated glutamine and asparagine as dynamic modifications.

### HPLC/MS/MS for quantification of NAD^+^/NADH

Cells pellets were resuspended in an ice-cold mixture of methanol/water (80/20, V/V). The mixtures were vortexed, sonicated (10 pulses, 50% intensity), and incubated on ice-bath for 30 min and the mixture was vortexed every 5 min during the incubation, followed by centrifugation at 10,000 rpm for 15 min at 4 °C. The supernatant was collected, and the extraction procedure was repeated one more time. The combined supernatant was dried on speed-Vac and then redissolved in 100 μl of 50/50 acetonitrile/water (V/V) containing 0.5 μg/ml of inosine-N15 as an internal standard. Samples and standard solutions were analyzed using an Agilent 1200 series HPLC system equipped with a binary pump and an autosampler (Agilent) was employed for HPLC separation. The HPLC separation was conducted on an Xbridge BEH amide HILIC column (2.1 × 150 mm, 2.5 μm, Waters) with a gradient elution. Mobile phase A was acetonitrile and mobile phase B consisted of aqueous ammonium acetate (10 mM)/acetonitrile (95/5, V/V) pH 9.0 adjusted with ammonia solution. The gradient started at 15% of B over 0 to 0.1 min, 0.1 to 9 min, 50% B; 9 to 13 min, 50% B and hold for 3 min, and then back to 15% B for column equilibrium for 17 min. The flow rate of the mobile phase was 150 μl/min and the injection volume was 5 μl. A hybrid triple quadrupole/linear ion trap 3200 QTRAP mass spectrometer (AB Sciex) using Analyst 1.4.2 software (https://sciex.com/products/software/analyst-software) for data acquisition and analysis. A multiple reaction monitoring scan mode under negative electrospray ionization was used to determine NAD^+^ and NADH. The transition ion pairs were 662 > 540 for NAD and 664 > 408 for NADH, with inosine-15N as the internal standard with a transition ion pair of 271 > 139. A five-point calibration NAD^+^ and NADH at concentrations of 0.5 to 10 μg/ml was constructed based on the peak area ratio of the standards/internal standards *versus* the concentration of the analyte. The concentration of NAD^+^ and NADH in cells was calculated with this calibration curve.

### Cell viability assay

Cell viability was assessed using the cell counting kit-8 assay (96992, Sigma). A549 (parental and Ndufs1-C92D-KI) and H1299 cells were seeded in 96-well plates at a density of 1 × 10^4^ cells per well and allowed to adhere overnight. The cells were treated with various concentrations of doxorubicin with or without pretreatment of MitoTam (200 nM, 12 h) for 24 h. After treatment, 10 μl of cell counting kit-8 solution was added to each well, followed by incubation at 37 °C for 1 h. Absorbance was measured at 450 nm using a microplate reader. IC50 values were calculated using GraphPad Prism software (version 8; https://www.graphpad.com) based on dose-response curves fitted to a four-parameter logistic regression model.

### Cell migration assay

Transwell migration assays were performed using 24-well plates with an 8-μm pore-size polycarbonate filter (ECM508, MilliporeSigma). Prior to the experiment, all media components were equilibrated to RT. The lower chambers were filled with 650 μl of complete DMEM (DMEM or RPMI-1640 with 10% FBS). In the upper chambers, we seeded 1 × 10^6^ cells (A549 or H1299) in 100 μl of serum-reduced medium (DMEM or RPMI-1640 with 2% FBS). After 24 h, the cells in the upper chamber (nonmigrated cells) of the Transwell were removed, and the cells migrated to the lower side of the membrane were fixed with 4% glutaraldehyde for 10 min at RT and stained with 0.2% crystal violet for 10 min at RT. Images were acquired using confocal microscopy (Carl Zeiss 780 LSM), and migrated cells were manually counted using ImageJ software, with background migration (cells migrating toward plain medium) subtracted from all experimental values.

### Plasmid transfections

Cells were seeded in 15 cm dishes at 70 to 80% confluence 24 h prior to transfection. Transfection was performed using Lipofectamine 3000 (Thermo Fisher Scientific), with 15 μg of DNA transfected into the cells, followed by incubation for 72 h. Plasmids encoding human WT or mutant Ndufs1 were obtained from GenScript and all sequencing and plasmid validation data are provided in the supplementary information section.

### siRNA transfection

Cells were seeded in 60 mm dishes at 70 to 80% confluence 24 h prior to transfection using Lipofectamine 3000 (Thermo Fisher Scientific). For transfection, 70 pmol of UQCRC2-targeting siRNA or scramble siRNA (both obtained from Santa Cruz Biotechnology) were added to the dishes. Cells were incubated for 72 h posttransfection before subsequent analysis. UQCRC2 siRNA (human, sc-72021) is a pool of 3 different siRNA duplexes: Sense: CCAUUUGCUGCGUCUUACAtt, Antisense: UGUAAGACGCAGCAAAUGGtt (sc-72021A); Sense: CUUGUAUUGUCCUGACUAUtt, Antisense: AUAGUCAGGACAAUACAAGtt (sc-72021B); Sense: CAACUCAGCAGCCAUUUGAtt, Antisense: UCAAAUGGCUGCUGAGUUGtt (sc-72021C). All sequences are provided in 5′ → 3′ orientation.

### Adenoviral transduction

A549 cells were transduced with GFP, WT-Ndufs1, or mutant-Ndufs1 adenoviruses obtained from GenScript. The cells were transduced at a multiplicity of infection of 50 for 48 h and then further incubated for 24 h with fresh complete medium.

## Statistics

Statistical analysis was performed using Prism (v8, GraphPad Prism). All assays were repeated at least in triplicate and the data are represented as mean ± SD, along with each individual data point. Data were tested for normality using Shapiro–Wilk. Two-tailed Student’s *t* test was used for comparisons between two groups, while one-way ANOVA was used for multiple comparisons with Dunnett’s or Turkey’s *post hoc* test. The details about statistic parameters are specified in the figure legends. Significance was considered at *p* < 0.05.

## Data availability

Data supporting this study are included within the article and/or supporting materials.

## Supporting information

This article contains supporting information.

## Conflict of interest

The authors declare that they have no conflicts of interest with the contents of this article.
