# Supplementary material for: Cysteine oxidation of a redox hub within complex I can facilitate electron transport chain supercomplex formation
Source: J Biol Chem. 2025 Aug 5;301(9):110555. doi: 10.1016/j.jbc.2025.110555 (PMC12409452; doi:10.1016/j.jbc.2025.110555)
Supplement: Supplementary Material [file mmc4.pdf]

Supplementary Figures

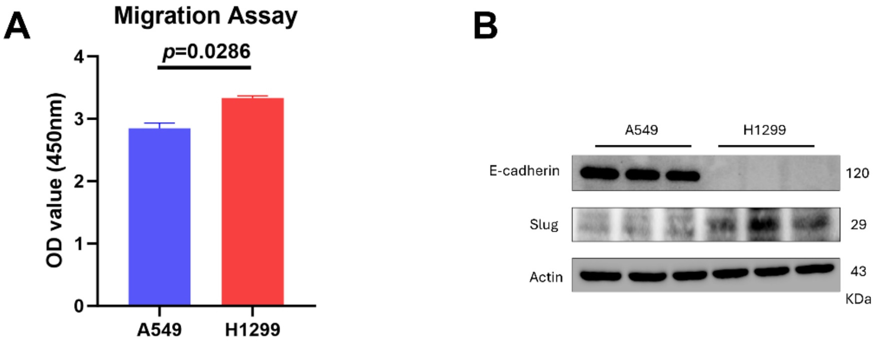

Fig S1: Transwell Migration and Immunoblot analysis for metastatic markers revealed enhanced metastatic features of H1299 cells compared to A549 cells. (A) Cell migration capacity was assessed using transwell migration assays in A549 and H1299 cell lines (n=3 independent experiments). Quantitative analysis demonstrated significantly higher migratory activity in H1299 cells (*p<0.05, Student's t-test). (B) Protein levels of epithelial-mesenchymal transition (EMT) markers were evaluated by immunoblots. H1299 cells exhibited decreased E-cadherin (epithelial marker) and increased Slug (mesenchymal marker) protein levels, compared to A549 cells (n=3 biological replicates). Actin was used as a loading control.

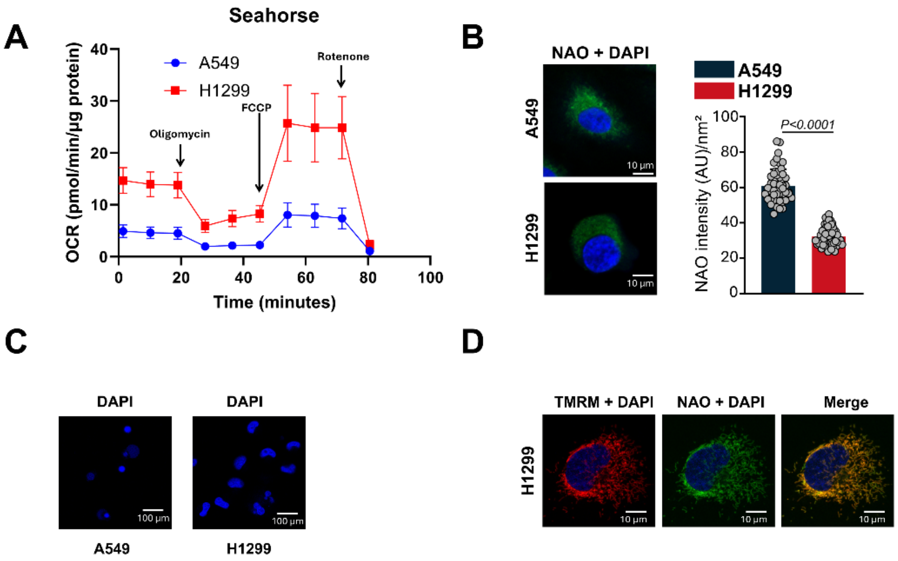

Fig S2: Mitochondrial mass and function in A549 and H1299 cells assessed by NAO staining and Seahorse assay. (A) Mitochondrial mass was evaluated using the cardiolipin-specific fluorescent dye acridine orange 10-nonyl bromide (NAO). Confocal microscopy revealed significantly reduced mitochondrial mass in H1299 cells compared to A549 cells (Student's t-test, n = 50 cells/group). Fluorescence signals were quantified in arbitrary units (AU). Staining: NAO (green, mitochondria), DAPI (blue, nuclei). Scale bar:10μm. (B) Co-localization of TMRM (tetramethylrhodamine methyl ester, red, mitochondrial membrane potential) and NAO (green, mitochondria) confirmed mitochondrial localization. DAPI (blue) was used for nuclear counterstaining. Scale bar:10μm. (C) Negative control with DAPI (blue, nuclei) only. Scale bar:10μm. (D) Mitochondrial respiration was measured using the Seahorse XF Analyzer, which showed higher oxygen consumption rate (OCR) in H1299, compared to A549 cells.

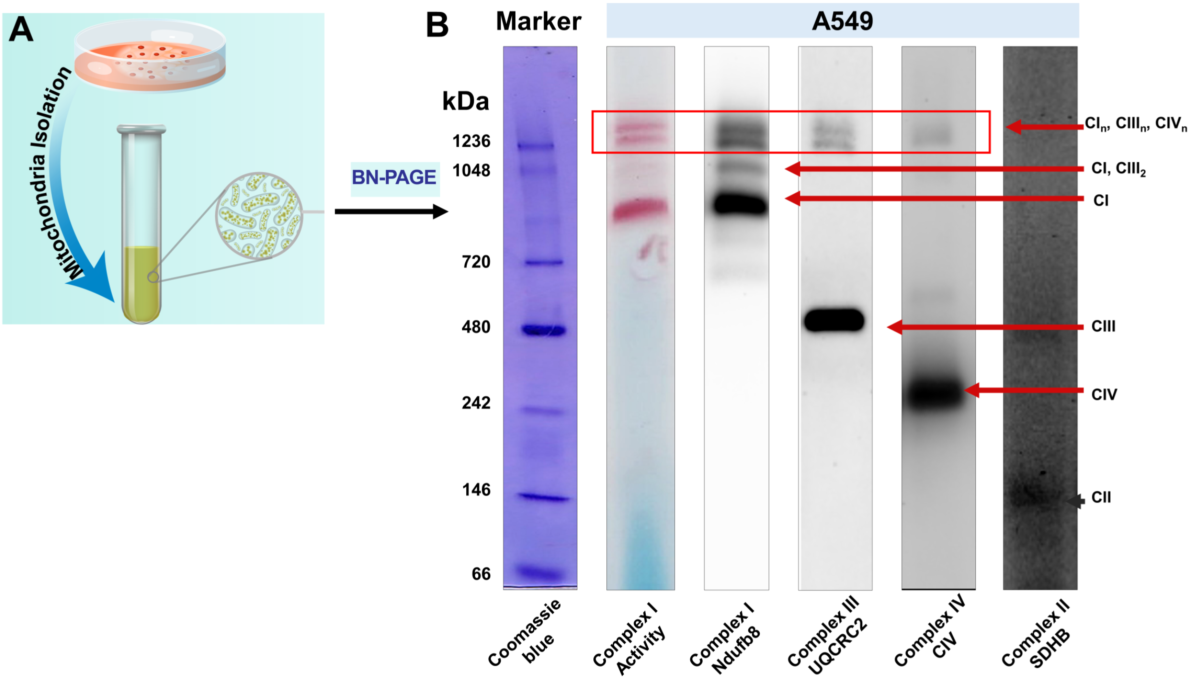

Fig S3: Mitochondrial protein analysis in A549 cells using BN-PAGE and SDS-PAGE. (A) Schematic of mitochondrial protein isolation procedure in human A549 cells, followed by Blue Native PAGE (BN-PAGE) and immunoblot analysis. Gel loaded with native unstained protein standard were stained with Coomassie brilliant blue R250.M. NADH dehydrogenase activity complex I were measured by in-gel complex I activity assay. Antibodies targeting NDUFB8 (complex I), SDHB (complex II), UQCRC2 (complex III) and CIV (complex IV), and were used to detect the respirasome

supercomplex and free complexes of mitochondrial protein from human A549 cells (B). BN, blue native.

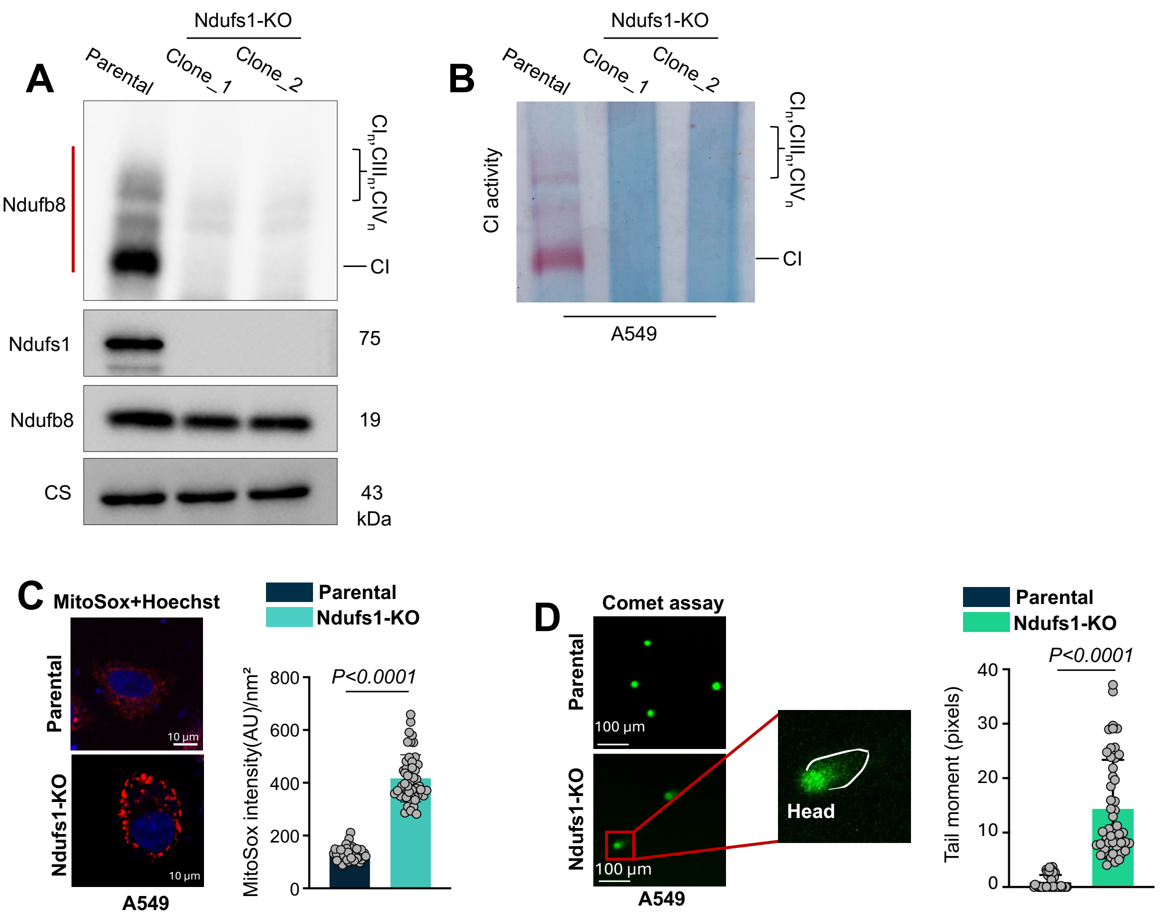

E

Non-failure human hearts (Proteomics)

Non-failure human heart / Modification Types	1(F/21Y)	2(F/63Y)	3(M/46Y)	4(M/45Y)
Sulfon	C78,C92	C75,C78	C75,C78	N/A
Sulfin	N/A	N/A	C75,C78	C75
Sulfen	N/A	N/A	C75	N/A

Fig S4: Identification of a redox hub within Ndufs1 that can directly facilitate the transformation towards the ETC respirasome. (A) Blue native PAGE (BN-PAGE) result demonstrated Ndufs1 knockout decreased mitochondrial supercomplex assembly (CI_n+$CIII_n$+CIV_n) in two different clones of Ndufs1 knockout A549 cells. Complex I (Ndufb8) and Citrate Synthase from SDS-PAGE were used as loading controls. (B) In-gel mitochondrial complex I activity was measured in parental A549 cells

and two different clones of Ndufs1 knockout A549 cells, showing decreased mitochondrial complex I activity. (C) Comparison of mitochondrial ROS in Ndufs1-KO (clone_1) and parental A549 cells using MitoSox, Student's t test on n=50 cells/group. AU, arbitrary unit. (D) A representative comet assay that shows DNA damage in A549 cells. The assay shows a notably larger tail moment in Ndufs1-KO cells (clone_1) compared to A549 parental cells. Student's t test on n=50 cells/group. (E) The cysteine oxidative modifications on Ndufs1 protein of non-failing heart tissues were identified using mass spectrometry analysis of Ndufs1 purification *via* co-IP. n=4. F, female; M, male; Y, years old. All the data are shown as mean ± S.D and p-values are presented in each panel. All the data are shown as mean ± S.D and p-values are presented in each panel.

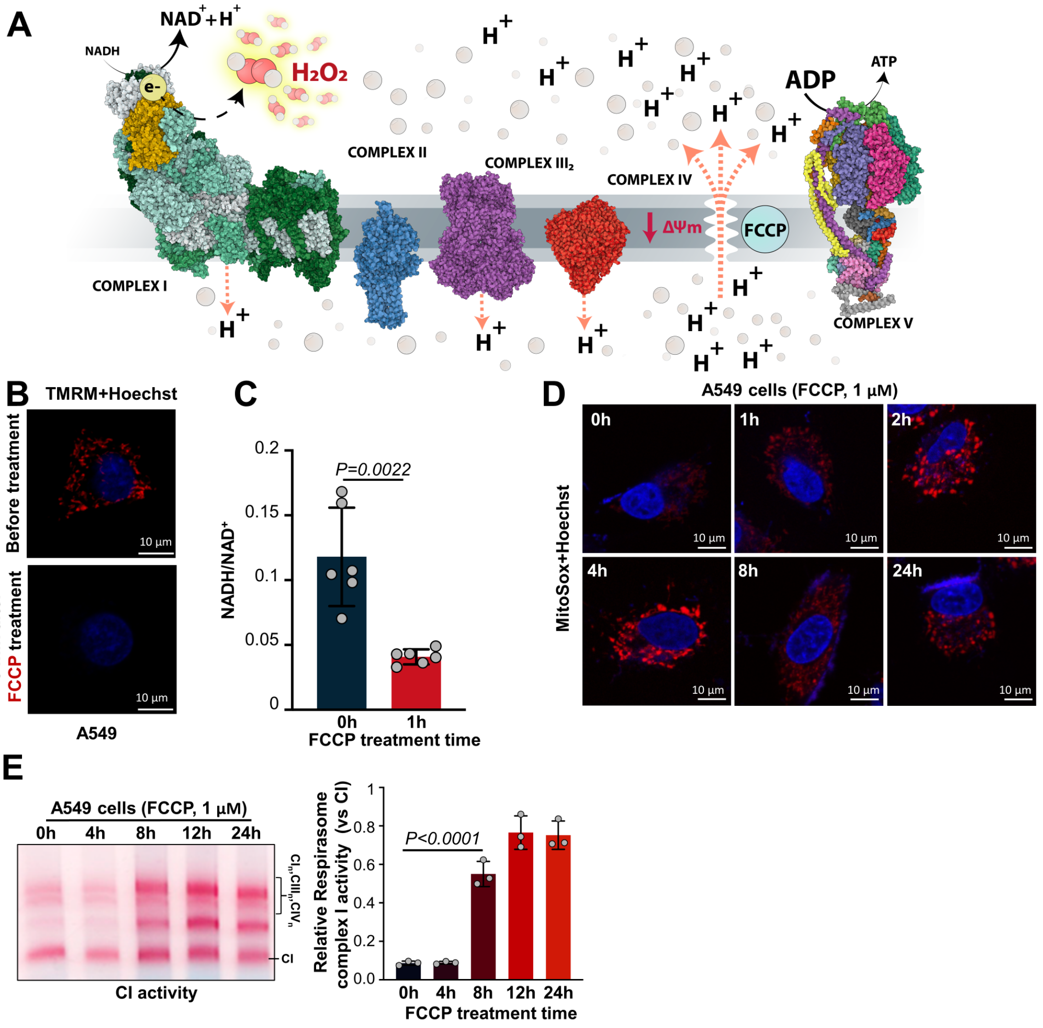

Fig S5: Assessment of mitochondrial functional and structural changes in human A549 Cells following FCCP treatment. (A) Schematic representation of the FCCP-mediated uncoupling of the

mitochondrial ETC from ATP synthase. (B) Representative confocal images of ΔΨm using Tetramethylrhodamine methyl ester (TMRM) in human A549 cells treated with 1μM FCCP for 5min. (C) Measurement of NADH and NAD+ by HPLC/MS/MS. The retention time from the chromatogram and ion transition pair were used for the NADH and NAD^+ identity. Student's t test on n=6 biologically independent replicates. (D) Representative confocal images of human A549 cells treated with 1μM FCCP (0-24h), using MitoSox. MitoSox (red) and Hoechst (blue). (E) In-gel assessment of mitochondrial complex I activity of mitochondrial proteins from A549 cells treated with 1μM FCCP (0-24h). FCCP, carbonyl cyanide-p-trifluoromethoxyphenylhydrazone. CI, complex I. One-way ANOVA with Dunnett's multiple comparisons test on n=3 biologically independent replicates. All data are shown as mean ± S.D and p-values are presented in each panel.

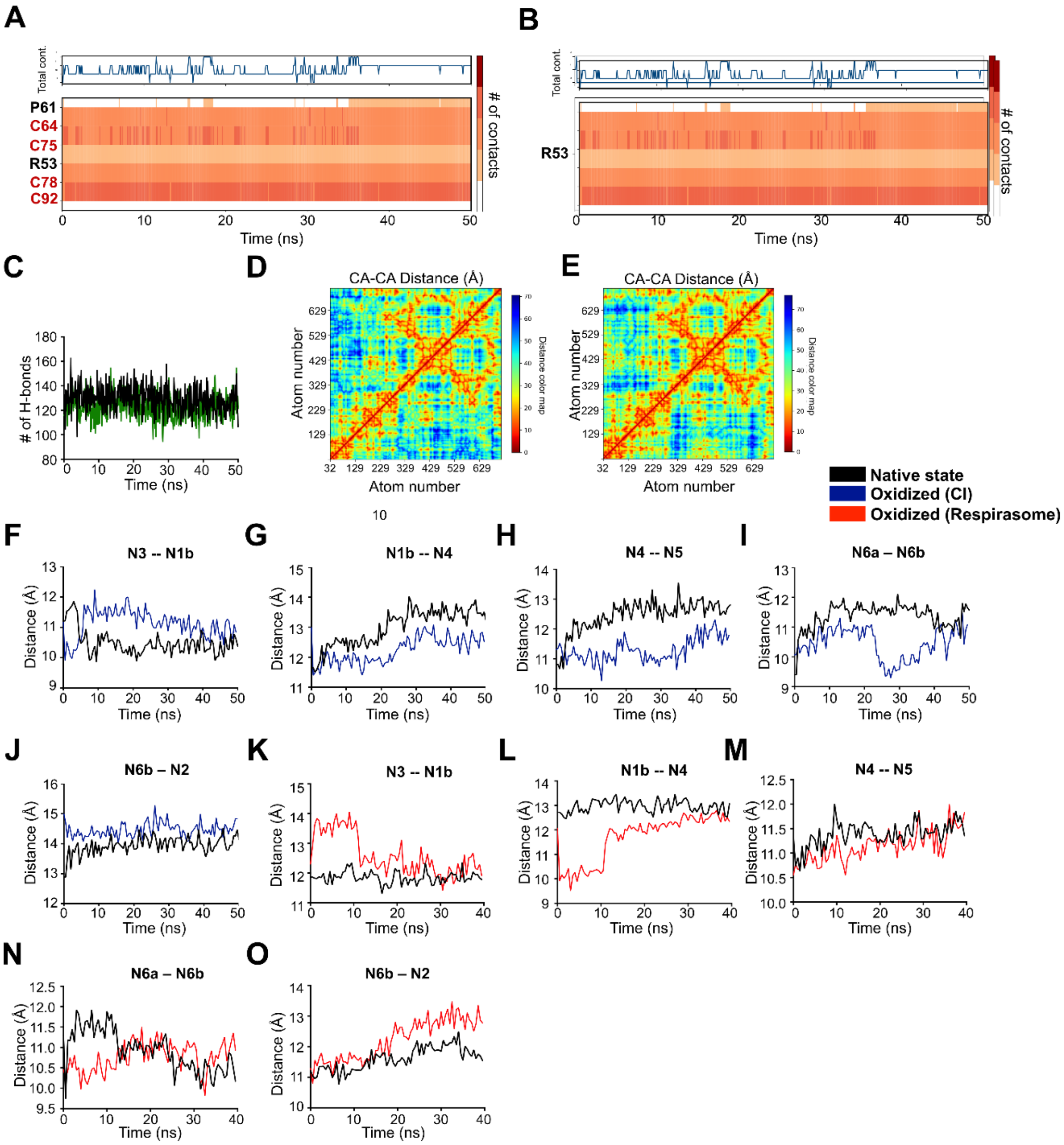

Fig S6: Comparative Interaction Dynamics and Cluster Spacing in Wildtype Versus Oxidized Ndufs1. (A&B) Chronological interaction profiles comparing contact patterns between native and oxidized Ndufs1 throughout the simulation timeline. (C) Comparative visualization of hydrogen bond interaction frequencies, delineating oxidized Ndufs1 (depicted with a green trajectory) against wildtype. (D&E) Interaction heatmap for alpha carbon proximity within Wildtype and oxidized Ndufs1, indicating residue-level contact differences. (F&J) Temporal analysis of inter-cluster spacing within Complex I's iron-sulfur regions, contrasting conditions of wildtype and oxidized Ndufs1. (K-O) Detailed temporal mapping of iron-sulfur cluster separations in the respirasome, juxtaposing wildtype with oxidized Ndufs1 throughout the MD simulation period.

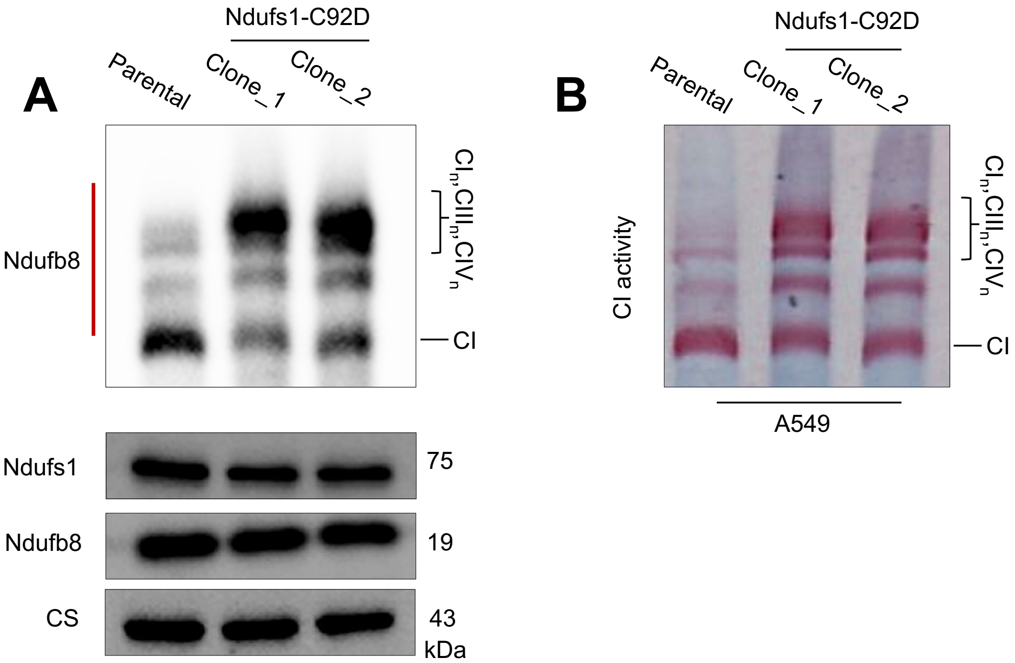

Fig S7: C92D-Ndufs1 mutant knock-in mitochondria from A549 cells have increased levels of the respirasome supercomplex and complex I activity. (A) Blue native PAGE (BN-PAGE) result showed increased mitochondrial supercomplex assembly (CI_n+$CIII_n$+CIV_n) in two different clones of C92D-Ndufs1 mutant knock-in cells, compared to the parental A549 cells. Complex I (Ndufs1 and Ndufb8) and Citrate Synthase from SDS-PAGE were used as loading controls. (B) In-gel mitochondrial complex I activity was measured in parental A549 cells and two different clones of C92D-Ndufs1 mutant knock-in cells, showing increased mitochondrial complex I activity.

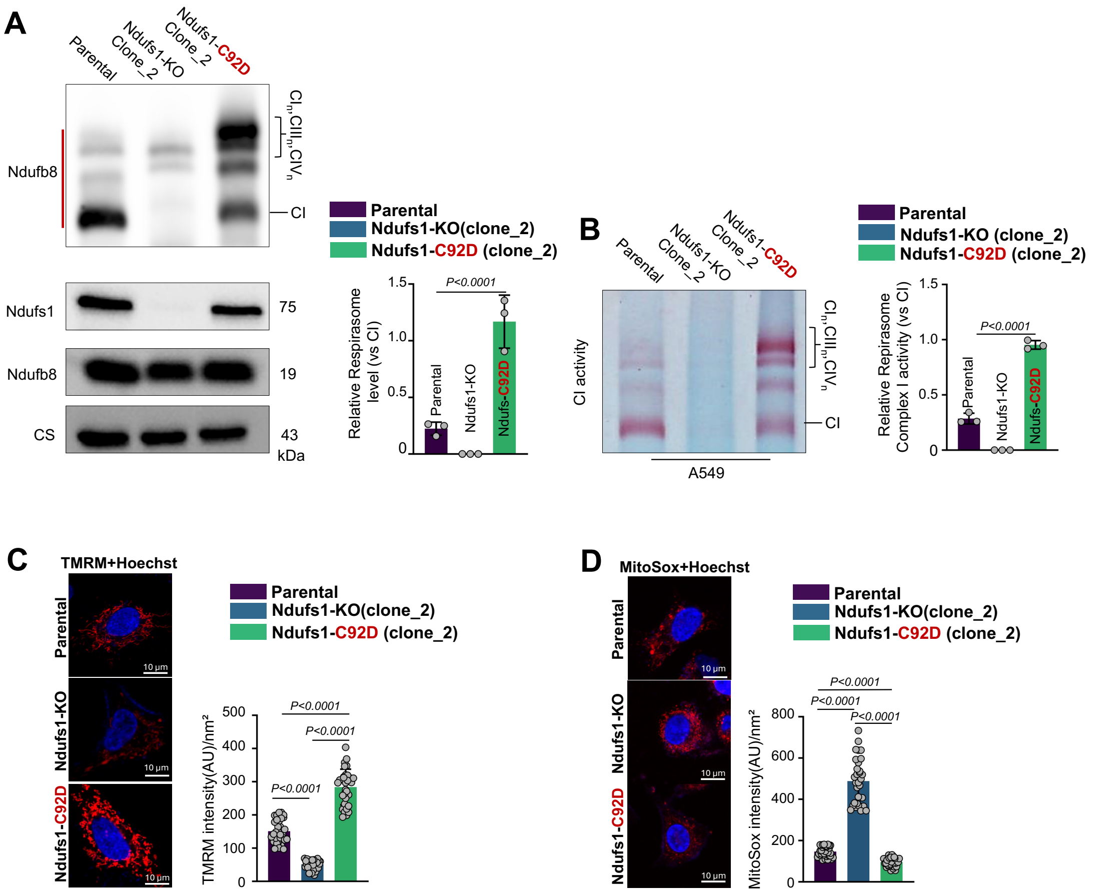

Fig S8. C92D mutant Ndufs1 knock-in mitochondria have increased levels of the respirasome supercomplex, complex I activity, mitochondrial membrane potential and decreased levels of mitochondrial ROS. (A) BN-PAGE and SDS-PAGE separation of mitochondrial proteins from human A549 cells (parental, Ndufs1 KO (clone2), and C92D-Ndufs1 knock-in (clone2)), followed by immunoblot analysis using antibodies against Ndufb8 for complex I and respirasome supercomplex in native samples and Ndufb8, Ndufs1, and Citrate Synthase (CS) in denatured samples. One-way ANOVA with Dunnett's multiple comparisons test on n=3 biologically independent replicates. (B) In-gel mitochondrial complex I activity was measured to determine the NADH oxidase activity ratio of the respirasome supercomplex to complex I. One-way ANOVA with Dunnett's multiple comparisons test on n=3 biologically independent replicates. (C) Mitochondrial membrane potential ($\Delta\Psi m$) assessment in A549 cells (parental, Ndufs1 KO (clone2), and C92D-Ndufs1 knock-in (clone2)) using Tetramethylrhodamine methyl ester (TMRM). Confocal images illustrate increased $\Delta\Psi m$ in C92D-Ndufs1 mutant cells and decreased $\Delta\Psi m$ in Ndufs1-KO cells compared to parental cells. One-way ANOVA with Tukey's multiple comparisons test on n=30 cells/group. AU, arbitrary unit. (D) Mitochondrial ROS were analyzed in A549 cells (parental, Ndufs1-KO (clone2), and

C92D-Ndufs1 knock-in (clone2)) labeled with MitoSox. Representative confocal images show lower mitochondrial ROS production in C92D-Ndufs1 mutant cells and higher mitochondrial ROS production in Ndufs1-KO cells compared to parental cells. One-way ANOVA with Tukey's multiple comparisons test on n=30 cells/group. AU, arbitrary unit.

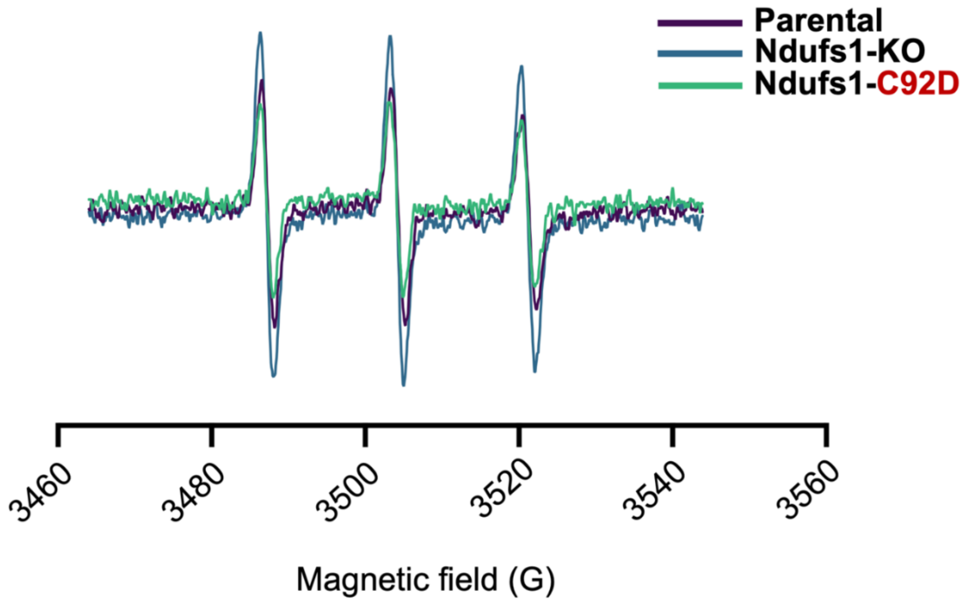

Fig S9: Mitochondrial superoxide levels in A549 parental, Ndufs1-C92D-mutant knock-in and Ndufs1-knockout cells. Direct measurement of mitochondrial superoxide production using electron paramagnetic resonance (EPR)-spectroscopy in A549 parental, Ndufs1-C92D-mutant knock-in cells and Ndufs1-knockout cells. AU, arbitrary units; G, gauss.

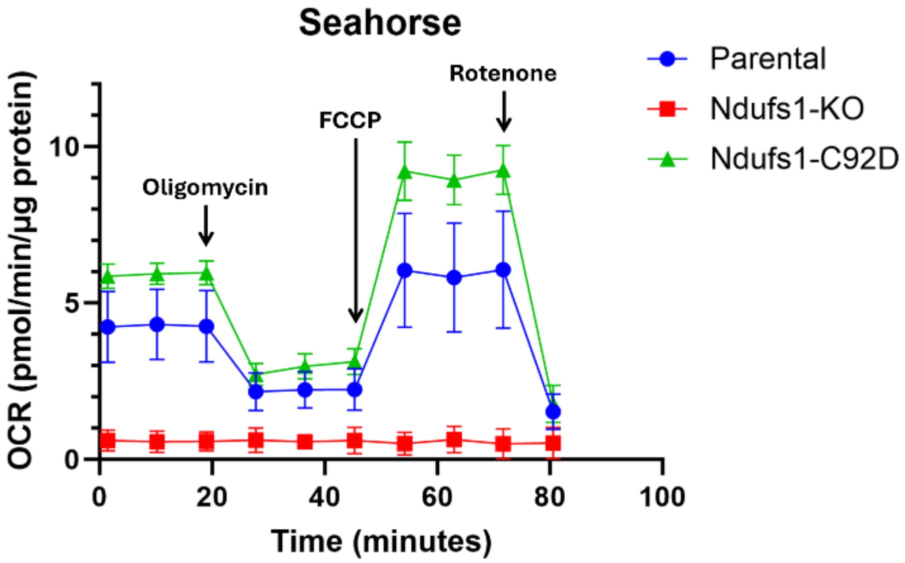

Fig S10: Mitochondrial respiration levels in A549 parental, Ndufs1-C92D-mutant knock-in and Ndufs1-knockout cells. Mitochondrial respiration was measured using the Seahorse XF Analyzer. The results showed significantly higher oxygen consumption rate (OCR) in Ndufs1-C92D mutant, compared to parental or Ndufs1 KO A549 cells.

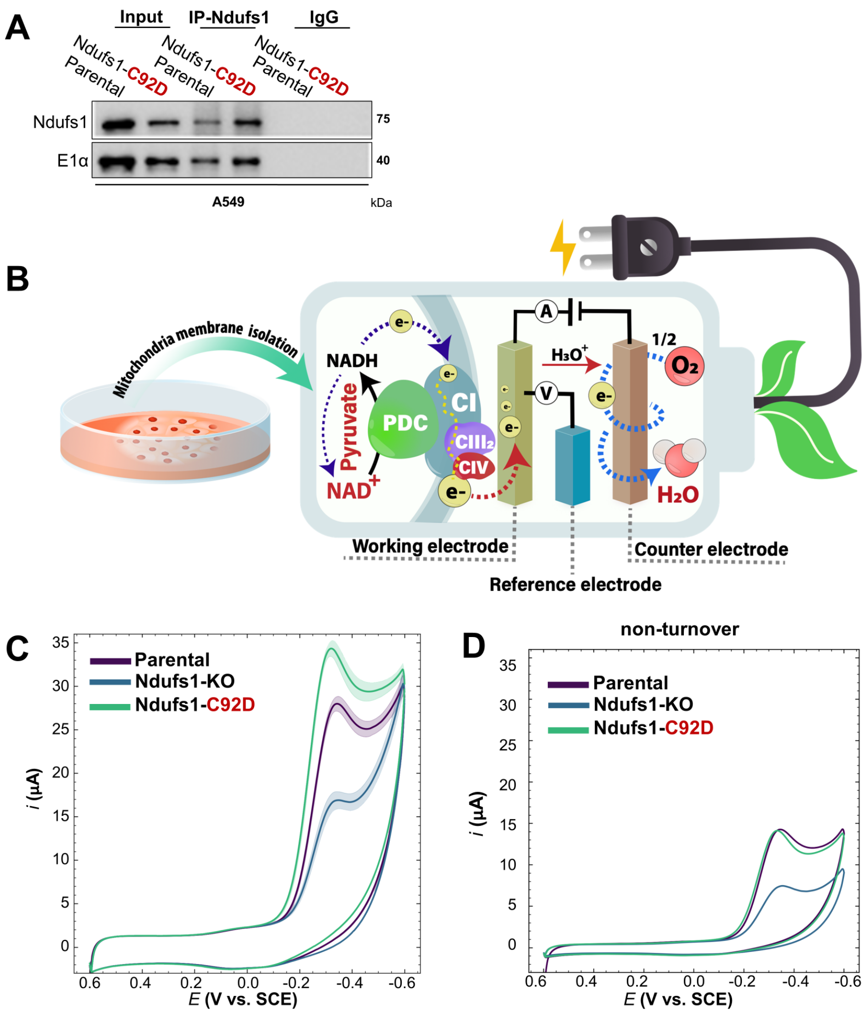

Fig S11. The ETC respirasome has efficient electron flow and could provide a technologic platform for a biobattery. (A) Immunoblot of pyruvate dehydrogenase (PDH) E1α on Ndufs1 in parental A549 cells and Ndufs1-C92D-mutant knockin A549 cells *via* co-IP against Ndufs1and PDH-E1α. (B) Schematic showing that a conventional three-electrode bioelectrochemical system with a saturated calomel electrode (SCE) as a reference was used for biofuel-cell battery test. (C) Cyclic voltammograms (first cycle) of carbon paper electrodes loaded with isolated mitochondrial membranes from C92D Ndufs1 knockin mutant, Ndufs1 KO and parental A549 cells in 2.5% Nafion 117. Voltammetry was performed in $NaNO_3$ (6M) with 1mM NAD^+ and 100 mM sodium pyruvate at 25 °C and 30 mV/s. Using a standard three-electrode cell consisting of the carbon paper working electrode, a saturated calomel electrode (SCE) reference, and a Pt mesh counter electrode. (D) Cyclic voltammograms (first cycle) of carbon paper electrodes ($0.25cm^2$) dropcast with purified mitochondria from C92D Ndufs1 mutant knock-in, Ndufs1 KO and parental A549 cells, in 2.5% Nafion 117. Voltammetry was performed in $NaNO_3$ (6M) in the absence of NAD^+ and sodium pyruvate at 25°C

and 30mV/s. Using a standard three-electrode cell consisting of the carbon paper working electrode, a saturated calomel electrode (SCE) reference, and a Pt mesh counter electrode, Standard deviation.

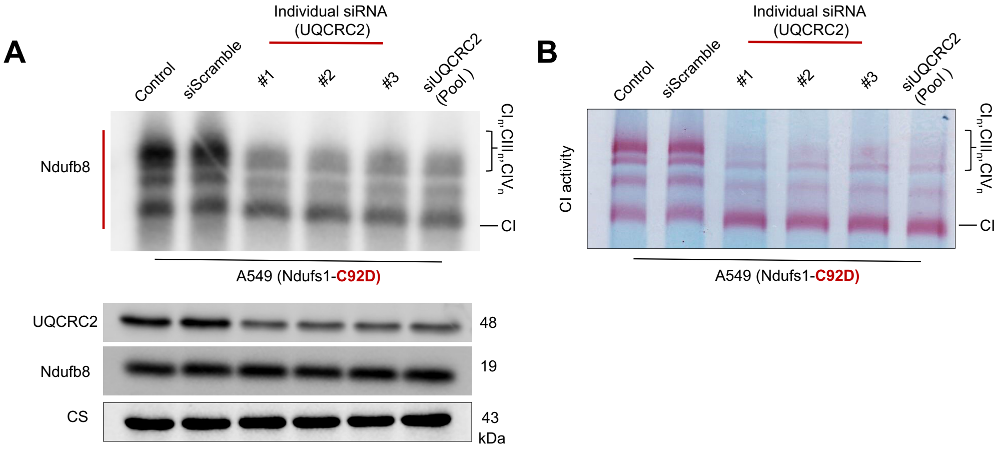

Fig S12: siRNA Knock-down of UQCRC2 decreased the levels of the respirasome supercomplex and complex I activity in C92D-Ndufs1 mutant knock-in A549 cells. (A) Blue native PAGE (BN-PAGE) result showed decreased mitochondrial supercomplex assembly (CI_n+$CIII_n$+CIV_n) in C92D-Ndufs1 mutant knock-in cells transfected with three different individual UQCRC2 siRNA and pooled UQCRC2 siRNA, compared to the parental A549 cells and scramble siRNA (different from the one used in Figure 4). Complex I (Ndufb8) and Citrate Synthase from SDS-PAGE were used as loading controls. (B) In-gel mitochondrial complex I activity was measured in in C92D-Ndufs1 mutant knock-in cells transfected with three different individual UQCRC2 siRNA and pooled UQCRC2 siRNA, compared to the parental A549 cells and scramble siRNA (different from the one in Figure 4), showing increased mitochondrial complex I activity.

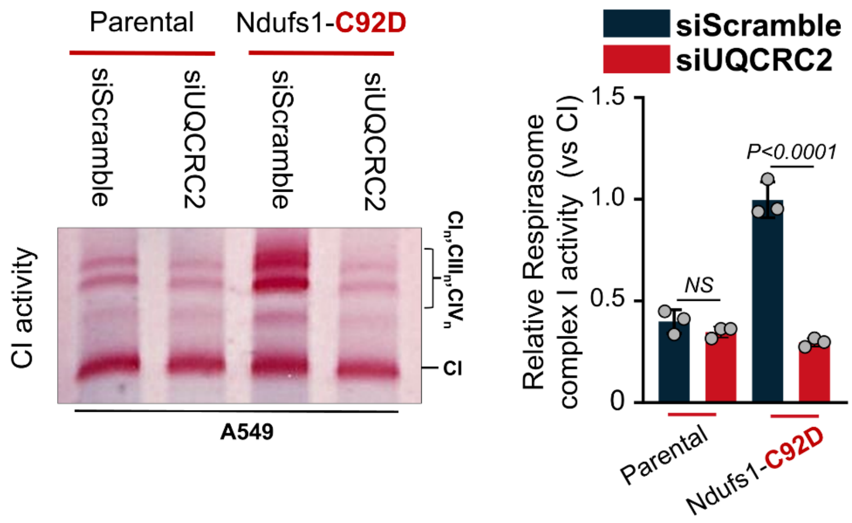

Fig S13: C92D mutant Ndufs1 knock-in mitochondria have increased levels of the respirasome supercomplex, where complex I activity and function are highly dependent on complex III. In-gel mitochondrial complex I activity was measured in parental A549 cells and C92D-Ndufs1 mutant knock-in cells transfected with UQCRC2 siRNA or scramble siRNA as control. Data are presented as mean values ± S.D of n = 6 biological replicas. Statistical significance was calculated using two-way ANOVA followed by Sidak's multiple comparison. p-values are presented in each panel. NS, non-significant.

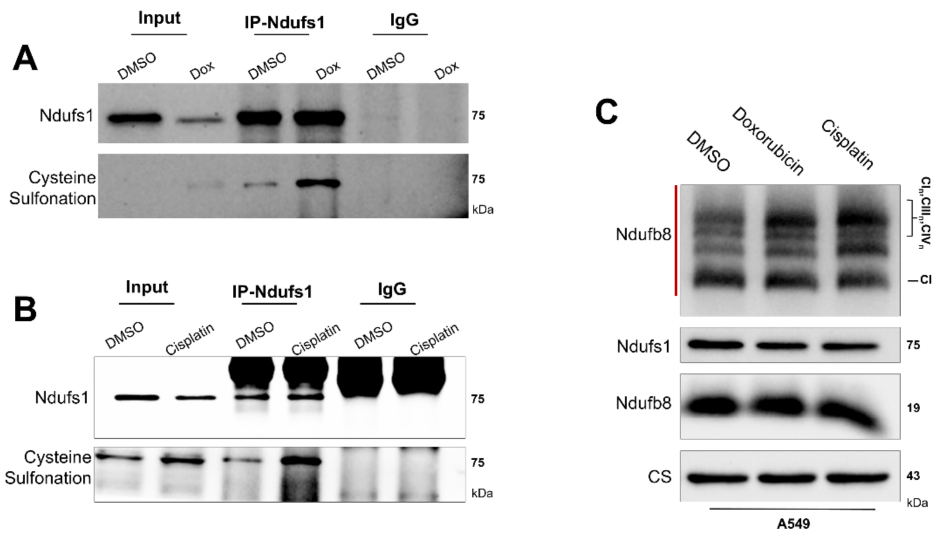

Figure S14. ROS-inducing chemotherapy agents can induce cysteine oxidation of Ndufs1 and increase mitochondrial supercomplex formation in A549 cells. (A, B) Co-immunoprecipitation

(Co-IP) followed by immunoblotting revealed increased sulfonation modification of Ndufs1 in A549 cells treated with doxorubicin (1µM) or cisplatin (20µM), compared to DMSO-treated controls. Blots were probed with an anti-Ndufs1 (loading control, top panels) and anti-cysteine sulfonation antibody (bottom panels). Data are representative of three independent experiments. (C) Blue native PAGE (BN-PAGE) analysis demonstrated elevated mitochondrial supercomplex assembly (CI_n+$CIII_n$+CIV_n) in A549 cells treated with doxorubicin or cisplatin, compared to DMSO controls. Complex I (Ndufs1 and Ndufb8) and Citrate Synthase from SDS-PAGE were used as loading controls.

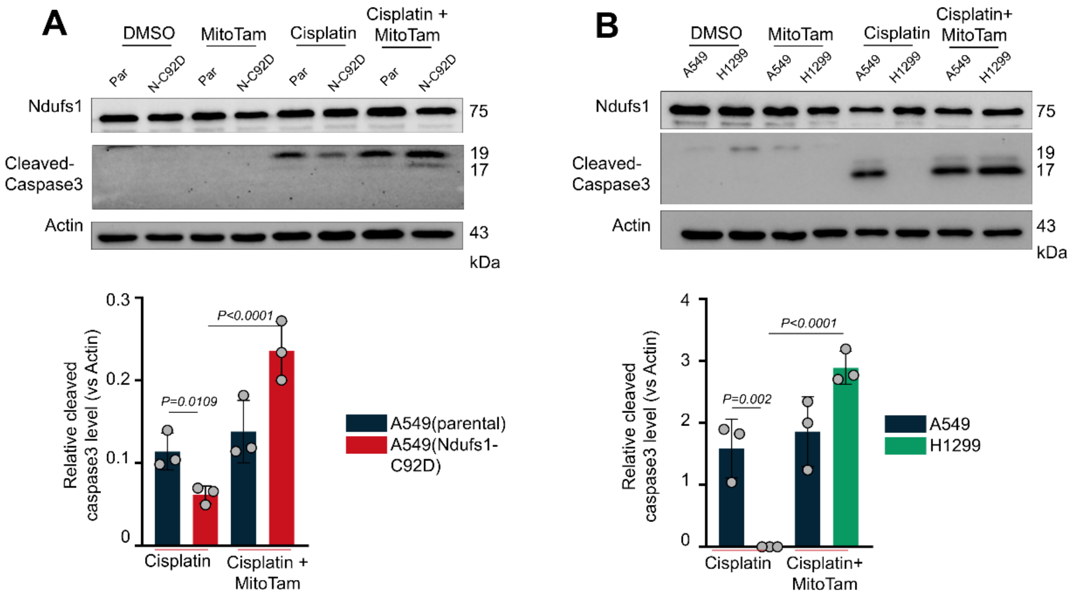

Fig S15: C92D mutant Ndufs1 knock-in A549 cells (N-C92D) and metastatic H1299 cells are more resistant to chemotherapy (Cisplatin)-mediated cell death, compared to parental A549 cells (Par). (A) Western blot analysis of cleaved caspase-3 expression in parental A549 cells and C92D mutant Ndufs1 knock-in A549 cells after DMSO, MitoTam (200nM, 24h), cisplatin (20µM, 24h) or cisplatin + MitoTam (combinatorial treatment), with Actin as a loading control. (B) Parallel analysis of cleaved caspase-3 in parental A549 cells versus metastatic H1299 cells under identical treatment conditions. Data are presented as mean ± S.D of three independent experiments. Statistical analysis was performed using two-way ANOVA followed by Sidak's multiple comparisons. p-values are presented in each panel.

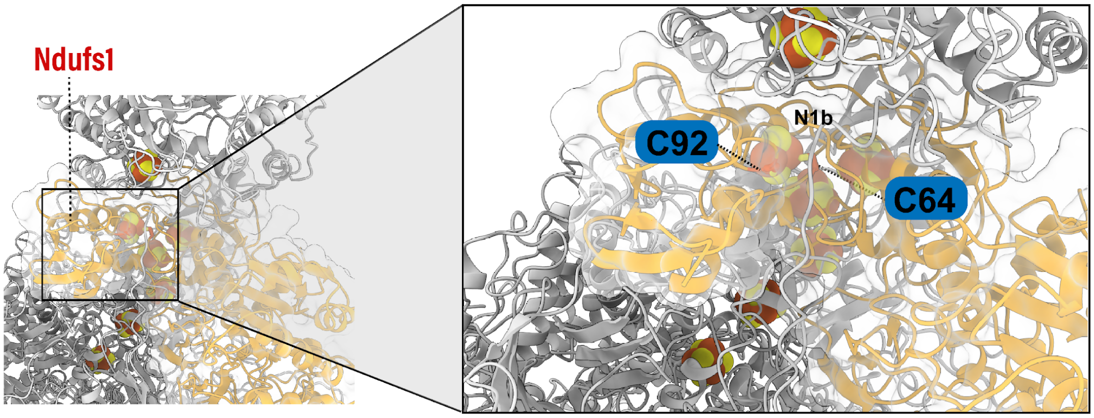

Fig S16: C92 exhibits greater oxidation susceptibility compared to C64. Residue C92 in Ndufs1 appears to be situated in a microenvironment conducive to oxidation due to its exposed position, while residue C64 is buried, indicating a reduced probability of oxidation.

Movie 1. Ndufs1 is positioned in an optimal location within complex I. Ndufs1 occupies a strategic location within complex I of the mitochondrial electron transport chain, having an essential role in facilitating the transfer of electrons from NADH to ubiquinone. This step is essential for generating the proton gradient and mitochondrial membrane potential ($\Delta\Psi m$) that can be utilized for ATP synthesis.

Movie 2. Dynamic Structural Changes in Ndufs1 Upon Cysteine Oxidation within the Redox Hub. Upon oxidation of cysteine residues (C64, C75, C78, C92) within Ndufs1 (isolated from complex I), a change in the spatial distance between N1b and N3 within Ndufs1, indicative of structural rearrangements induced by the oxidation event. The visualization provides insights into the dynamic behavior of Ndufs1 in response to oxidative modifications, shedding light on potential mechanisms underlying the functional regulation of complex I.

Movie 3. Restoring Efficient Electron Flow in Complex I through Respirasome Formation after Cysteine Oxidation of the Redox Hub. Upon oxidation of cysteine residues (C64, C75, C78, C92) of Ndufs1 within complex I, the distance between the N5 and N6A clusters exceeds 15 Å, disrupting efficient electron flow within the electron transport chain (ETC). However, upon formation of respirasomes, this distance is restored and stabilized at approximately 14 Å, re-establishing electron flow within the ETC.
